# FomA‐Containing Outer Membrane Vesicles of *Fusobacterium Nucleatum* Facilitate Bladder Cancer Lymphatic Metastasis via IL‐6‐Dependent M2b Macrophage Polarization

**DOI:** 10.1002/advs.202523256

**Published:** 2026-02-13

**Authors:** Wentai Shangguan, Weijia Li, Wenxue Huang, Jilin Wu, Yao Yu, Yiyao Huang, Lin Yang, Ming Xie, Qishen Yang, Jun Zheng, Yuexuan Zhu, Qi Sun, Biao Li, Leqian Li, Zongwei Wang, Jie Zhao, Peng Wu, Bisheng Cheng

**Affiliations:** ^1^ Department of Urology Nanfang Hospital,Southern Medical University Guangzhou China; ^2^ Department of Urology Second Affiliated Hospital of Naval Medical University Shanghai China; ^3^ Department of Urology Peking University People's Hospital Beijing P. R. China; ^4^ School of Medicine Shenzhen Campus of Sun Yat‐Sen University Sun Yat‐sen University Shenzhen Guangdong P. R. China; ^5^ Department of Surgery Division of Urology Beth Israel Deaconess Medical Center Harvard Medical School Boston Boston Massachusetts USA; ^6^ NMPA Key Laboratory For Research and Evaluation of Drug Metabolism Guangdong Provincial Key Laboratory of New Drug Screening School of Pharmaceutical Sciences Southern Medical University Guangzhou P. R. China; ^7^ Department of Clinical Oncology The University of Hong Kong‐Shenzhen Hospital Shenzhen P. R. China; ^8^ Department of Clinical Oncology Li Ka Shing Faculty of Medicine The University of Hong Kong Hong Kong SAR P. R. China

**Keywords:** bladder cancer, fomA, IL‐6, fusobacterium nucleatum, lymph node metastasis, M2b macrophage, vegf‐C

## Abstract

Outer membrane vesicles (OMVs) derived from the microbiota have emerged as key modulators of tumor progression and the immune microenvironment. However, the role of urinary microbiota and their associated OMVs proteins in the metastatic processes of bladder cancer (BCa) remains insufficiently understood. In this study, we investigated the impact of urinary microbiota on BCa progression and identified potential biomarkers within the urinary microbiome. We identified *Fusobacterium nucleatum* (*F. nucleatum*) as a predominant member of the urinary microbiota. Proteomic analysis of *F. nucleatum* OMVs revealed the outer membrane protein FomA as the most abundant component. A FomA‐deficient *F. nucleatum* mutant strain was generated to assess the relationship between FomA and lymph node (LN) metastasis. Mechanistically, FomA‐containing OMVs directly engage Toll‐like receptor 2 (TLR2), triggering the NF‐κB signaling pathway and upregulating interleukin‐6 (IL‐6) expression. Elevated IL‐6 induces M2b macrophage polarization, which subsequently promotes the release of VEGF‐C to facilitate LN metastasis. Furthermore, we identified pinocembrin, a natural flavonoid, as a potent inhibitor of the FomA–TLR2 interaction, effectively suppressing BCa progression. Collectively, our findings uncover a previously unrecognized microbiota‐driven mechanism by which *F. nucleatum*‐derived OMVs reprogram the tumor immune microenvironment toward a pro‐metastatic state and highlight FomA as a promising therapeutic target.

## Graphical abstract

Urinary *Fusobacterium nucleatum*–derived outer membrane vesicles are shown to promote bladder cancer lymphatic metastasis. The vesicle protein FomA activates TLR2/NF‐κB signaling in tumor cells, induces IL‐6 secretion, and drives M2b macrophage polarization and VEGF‐C–dependent lymphangiogenesis, revealing a microbiota‐driven mechanism linking tumor cells and the immune microenvironment.

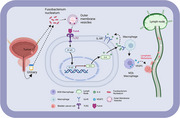

## 1 Introduction

Bladder cancer (BCa) is a prevalent malignancy of the urogenital system, commonly classified into non‐muscle‐invasive BCa (NMIBC) and muscle‐invasive BCa (MIBC) [1, 2]. Muscle‐invasive bladder cancer (MIBC) is an aggressive disease with a high risk of progression and mortality. A defining feature of advanced MIBC is its strong propensity for lymph node (LN) metastasis, which is a major determinant of patient survival. LN metastasis is not driven solely by tumor cell–intrinsic migratory and invasive behavior but instead represents a coordinated, multistep process shaped by interactions among tumor cells, the lymphatic system, and the tumor immune microenvironment, with tumor‐associated macrophages (TAMs) acting as key regulators [3, 4, 5]. Recent advances have revealed that the urinary tract, once considered sterile, harbors a complex microbiome. Dysbiosis within microbiota has been implicated in various urological diseases [6, 7, 8, 9]. Notably, the bladder serves as a reservoir for urine and is constantly exposed to urinary microbiota. Although the influence of microbiota on cancer has gained increasing recognition, the specific roles of urinary microbiomes and tumor‐resident microbiomes in bladder cancer progression remain largely undefined.

*Fusobacterium nucleatum* (*F. nucleatum or F. n*), a Gram‐negative anaerobic bacterium, has been associated with the progression of cancers, including colorectal, breast, cervical and oral cancer [10, 11, 12, 13]. The present studies indicate a potential involvement of *F. nucleatum* in BCa, with its presence detected in both the urine and tumor tissues of BCa patients [14]. A key mechanism by which *F. nucleatum* influences tumor progression is through the release of outer membrane vesicles (OMVs). OMVs are nano‐sized vesicles (20–300 nm in diameter) derived from the bacterial outer membrane. OMVs play essential roles in bacterial physiology and pathogenesis, including toxin delivery, metabolite secretion, bacteriophage infection, cell‐to‐cell communication, and immunomodulation [15]. OMVs derived from *F. nucleatum* are enriched in specific outer membrane proteins (OMPs), which contribute to vesicle formation, stability, and interactions with host cells [16, 17]. OMVs contain functional proteins such as *Fusobacterium* outer membrane protein A (FomA). FomA facilitates bacterial adhesion and invasion and has been shown to interact with host cells via Toll‐like receptor 2 (TLR2), triggering inflammatory responses [18, 19]. The chemokine, including Interleukin 6 (IL‐6), produced by different types of tumors has also shown effects on metastasis and macrophage polarization [20, 21, 22]. Tumor‐derived IL‐6 recruits different subsets of immune cells, including TAMs, which contribute to the progression of cancer cells and immune response evasion [23, 24, 25].

TAMs exhibit remarkable plasticity within the tumor microenvironment. Their functional polarization profoundly influences tumor progression [26] and has been linked to LN metastasis [27]. Microbiota have been implicated in modulating cytokine and chemokine production, thereby influencing TAMs polarization [28, 29, 30]. Recent classifications further subdivide M2 macrophages into M2a, M2b, M2c, and M2d subtypes [31]. Among them, M2b macrophages are uniquely induced by the co‐stimulation of immune complexes and TLR ligands, and are characterized by dual immunomodulatory functions of simultaneously secreting inflammatory cytokines and promoting immunosuppression [32]. However, the immune activation or inhibition function of FomA in the tumor microenvironment is unknown, and the interaction mechanism between FomA, TLR2, and upregulation of IL‐6 still needs to be verified.

To address the existing knowledge gap, we investigated the impact of urinary microbiota‐derived OMVs on BCa and their underlying mechanisms. We showed that FomA‐containing OMVs bind to TLR2 on BCa cells, leading to increased IL‐6 transcription, which in turn drives M2b macrophage polarization, enhances VEGF‐C secretion, and promotes lymph node metastasis in BCa. Additionally, we identified pinocembrin as a compound that targets the FomA‐TLR2 binding interface and significantly inhibits FomA‐driven tumor metastasis. These findings provide new insights into the urinary microbiota‐host interaction, positioning FomA‐containing OMVs from *Fusobacterium nucleatum* as a promising prognostic marker and therapeutic target in BCa.

## 2 Results

### 2.1 The Abundance of *F. nucleatum* is Associated with the Lymphatic Metastasis of Bladder Cancer

**FIGURE 1:**
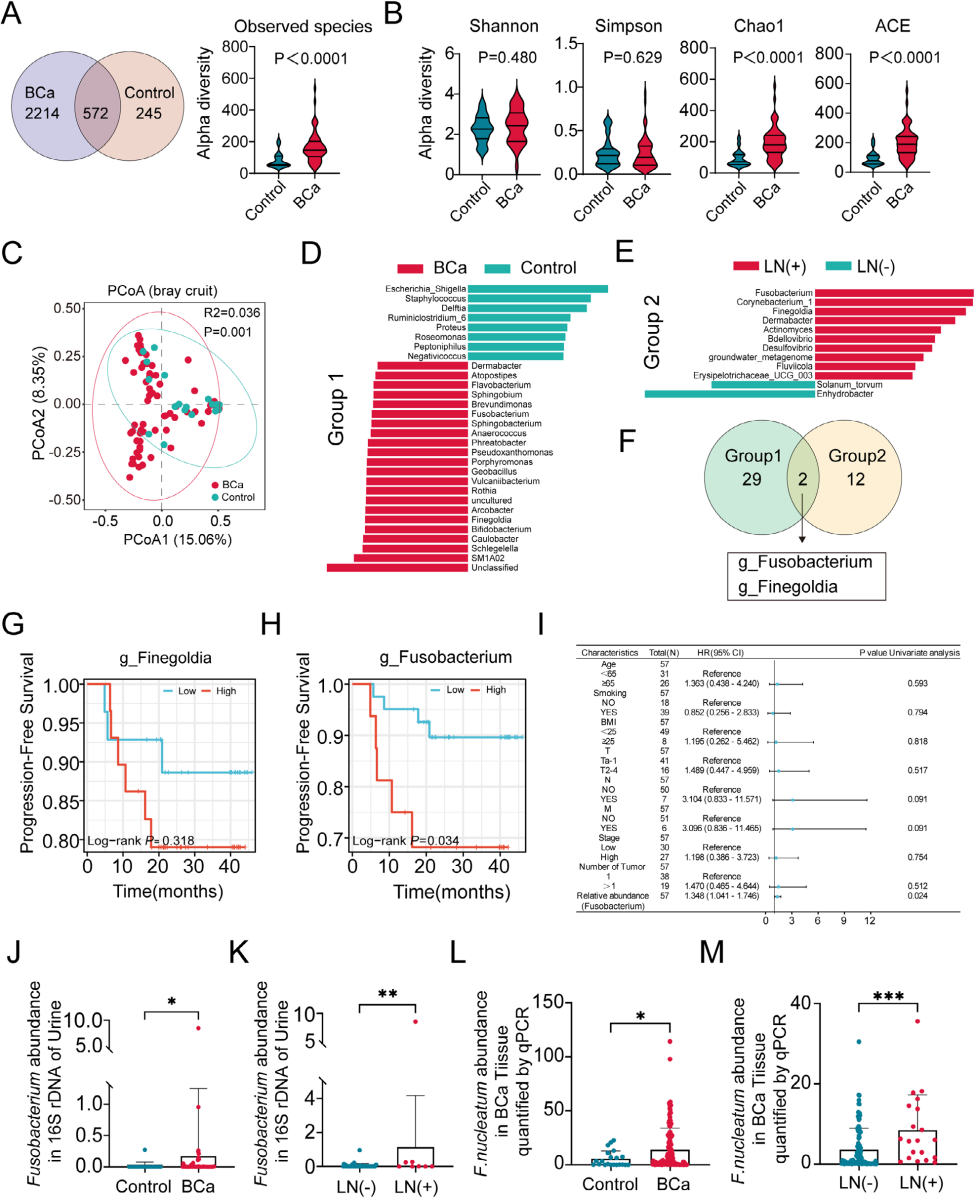
*Fusobacterium nucleatum* abundance is associated with lymph node metastasis in bladder cancer. (A) A Venn diagram and the species difference. (B) Alpha diversity analysis of urinary microbiota, including Shannon, Simpson, Chao1, and ACE indices. (C) Beta diversity of urinary microbiota assessed by principal coordinates analysis (PCoA). (D) LEfSe analysis of urinary microbiota at the genus level comparing Control and BCa groups. (E) LEfSe analysis of urinary microbiota at the genus level comparing LN(‐) and LN(+) groups. (F) Overlapping differential taxa were identified in both groups. (G,H) Kaplan‒Meier curves of progression‐free survival in BCa patients stratified by high vs. low relative abundance of *Fusobacterium* and *Finegoldia*. (I) Univariate analysis of the association between clinical characteristics and *Fusobacterium* abundance in BCa patients. (J) Relative abundance of *Fusobacterium* in urine samples from the Control and BCa groups. (K) Relative abundance of *Fusobacterium* in urine samples from LN(‐) and LN(+) groups. (L) *F. n* relative abundance in BCa vs. adjacent normal tissues. (M) *F. n* relative abundance in BCa tissues of the LN(‐) group and the LN(+) group. Data are presented as mean±SEM, ^*^ *p* < 0.05, ^**^ *p* < 0.01, ^***^ *p* < 0.001; ns: not significant. *F. nucleatum* or *F. n: Fusobacterium nucleatum*.

To investigate the differences in urinary microbiota composition between patients with bladder cancer (BCa, 64 samples) and healthy controls (NC, n = 18), we performed 16S rDNA sequencing. Venn diagram analysis revealed 572 shared bacterial taxa between the two groups, while overall microbial richness was significantly higher in the BCa group, with a marked increase in species richness (Figure 1A). In the α‐diversity analysis, no significant differences were observed in the Shannon and Simpson indices between the BCa and NC groups (*p* > 0.05, Figure 1B). In the contrast, the Chao1 and Ace indices were significantly higher in the BCa group (*p* < 0.0001, Figure 1B), suggesting increased microbial richness associated with BCa. β‐diversity analysis was performed using principal coordinate analysis (PCoA) based on different distance metrics. Visualization of β‐diversity revealed distinct clustering patterns between the BCa and Control groups (Figure 1C), and Adonis testing confirmed that the microbial community structures were significantly different between the two groups (*p* < 0.05). To identify the specific bacterial taxa responsible for these differences, we performed a linear discriminant analysis effect size (LEfSe) analysis on Group 1, defined by BCa and NC samples. *Fusobacterium* was identified as one of the most significantly enriched genera in the BCa group (LDA > 2.0, *p* < 0.05; Figure 1D). Given the clinical importance of lymph node (LN) metastasis in BCa progression, we next conducted LEfSe analysis on Group 2, stratified by LN metastasis status within the BCa cohort. Notably, *Fusobacterium* emerged as the most significantly enriched genus in lymph node‐positive (LN(+)) groups compared with LN‐negative (LN(‐)) groups (Figure 1E). Overlapping differential taxa identified in both groups contain *Fusobacterium* and *Finegoldia* (Figure 1F). Kaplan‒Meier curves analysis further demonstrated that elevated *Fusobacterium* abundance was significantly associated with reduced overall survival in BCa patients, whereas *Finegoldia* showed no prognostic relevance (Figure 1G,H). Although multivariate analysis did not retain a significant association (Figure S1), univariate analysis revealed significant variation in the relative abundance of *Fusobacterium* across clinical samples (Figure 1I). The relative abundance of *Fusobacterium* was markedly higher in the BCa group (Fig. 1J). Notably, when stratified by LN status, *Fusobacterium nucleatum* abundance was significantly higher in the LN(+) subgroup than in the LN(‐) subgroup (Figure 1K).  To validate these findings at the tissue level, species‐specific identification of *F. nucleatum* was confirmed by 5R 16S rDNA sequencing. And qPCR analysis demonstrated significantly higher abundance of *F. nucleatum* in bladder tumor tissues compared with adjacent normal tissues (Figure 1L). Furthermore, within tumor samples, *F. nucleatum* abundance was significantly elevated in patients with LN(+) groups relative to LN(‐) groups (Figure 1M). Collectively, these results identify *F. nucleatum* as a key urinary and tumor‐associated bacterium in BCa and suggest its potential clinical significance in the lymphatic metastasis of BCa.

### 2.2 Outer Membrane Vesicles of *F. nucleatum* Promote Bladder Cancer Metastasis

**FIGURE 2:**
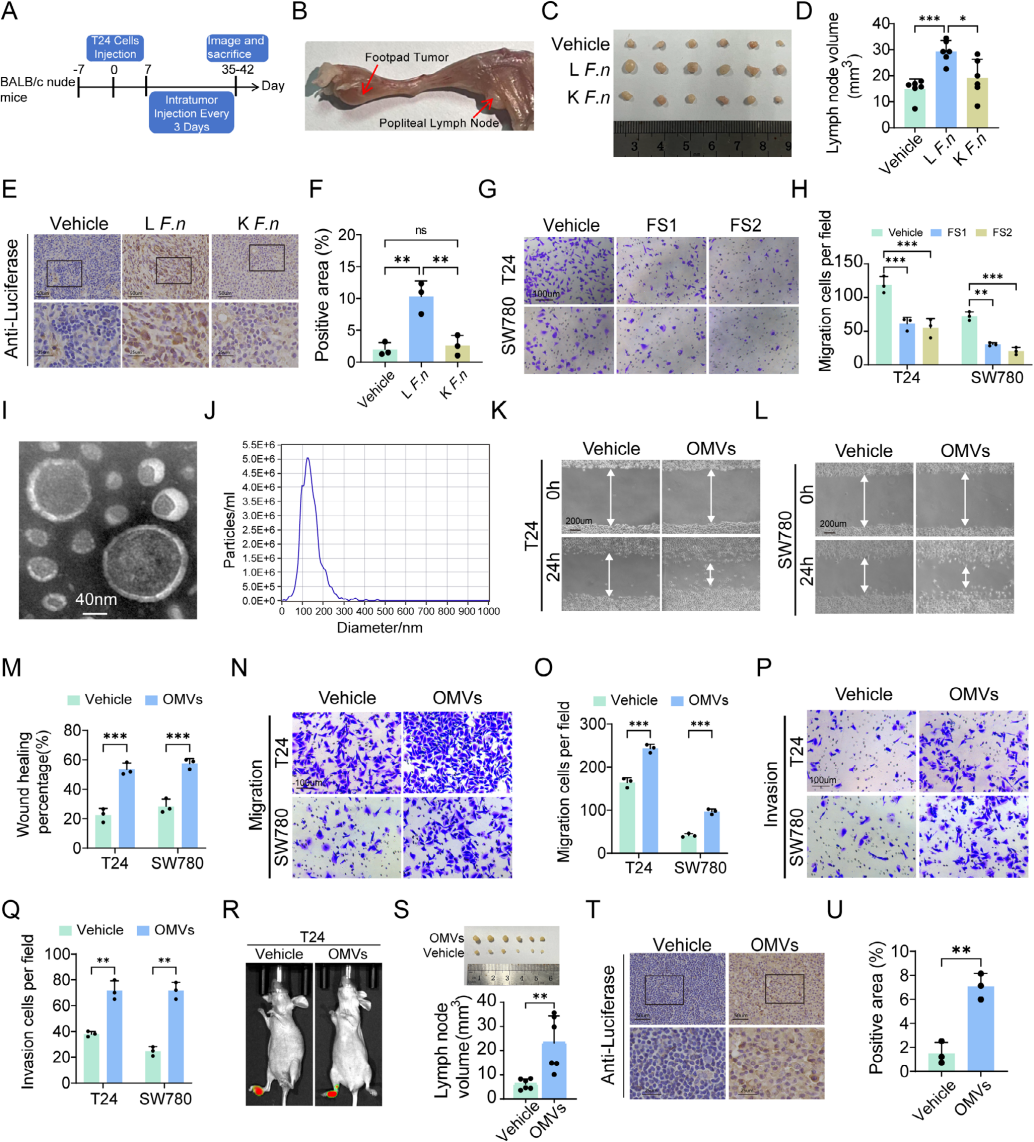
*Fusobacterium nucleatum–*derived OMVs promote migration, invasion, and lymphatic metastasis. (A) Schematic illustration of the lymph node metastasis model. (B) Representative bioluminescence imaging of LN metastasis. (C) Representative image of dissected popliteal LNs (n=6). (D) Quantification of popliteal LNs volumes. (E,F) Representative immunohistochemical (IHC) images and quantitative analysis of luciferase expression in popliteal LNs. (G,H) Transwell migration assay images and quantitative analysis of BCa cells treated with FS. (I) Transmission electron microscopy of purified *F. n* OMVs. (J) Nanoparticle tracking analysis of *F. n* OMVs. (K–M) Wound‐healing assay images and quantification of T24 and SW780 BCa cells under different treatment conditions. (N,O) Transwell migration assay images and quantification of migrated BCa cells. (P,Q) Transwell invasion assay images and quantification of invaded BCa cells. (R) Representative bioluminescence imaging. (S) Representative image of dissected popliteal LNs and quantitative analysis of LNs volumes (n=6). (T,U) Representative images and quantitative analysis of IHC staining. Data are presented as mean±SEM, ^*^ *p* < 0.05, ^**^ *p* < 0.01, ^***^ *p* < 0.001; ns, not significant. OMVs: outer membrane vesicles. L *F. n*: Live *F. nucleatum*. K *F. n*: heat‐killed *F. nucleatum*.

To investigate the role of *F. nucleatum* in BCa lymph node metastasis, we established a popliteal lymph node metastasis model using T24 cells expressing firefly luciferase in nude mice (Figure 2A). Mice received intratumor injections of PBS, live *F. n* (L *F. n*), or heat‐killed *F. n* (K *F. n*). Gross examination revealed marked enlargement of popliteal lymph nodes in the L *F. n* group (Figure 2B). And quantitative analysis confirmed that lymph nodes from L *F. n* were significantly larger than those from PBS or K *F. n* group (Figure 2C,D). BCa cells in the model were labelled with luciferase. Immunohistochemical (IHC) staining for luciferase showed a marked increase in luciferase‐positive cells within lymph nodes from mice treated with the OMVs group compared with PBS controls (Figure 2E,F). To identify the bacterial components responsible for this pro‐metastatic effect, we hypothesized that either secreted metabolites in the supernatant or outer membrane vesicles (OMVs) might be the functional effectors. Following 48 h of *F. n* culture, the medium was subjected to ultracentrifugation to isolate the *F. n* supernatant (FS) and OMVs (Figure S2C). Treatment of BCa cells with filtered FS disrupted cell attachment and inhibited cell proliferation in a dose‐dependent manner (Figure S2A,B), without inducing a significant migratory response (Figure 2G,H). In contrast, inhibition of OMVs secretion using GW4869 did not affect *F. n* proliferation (Figure S2D), but significantly reduced vesicle production (Figure S2E). OMVs isolated from the GW4869‐treated bacteria exhibited a markedly diminished capacity to promote cell migration, suggesting that OMVs are the primary mediators of *F. nucleatum*‐induced metastatic potential. The vesicles were characterized using transmission electron microscopy (TEM) and nanoparticle tracking analysis (NTA) (Figure 2I,J). In scratch wound assays, *F. n* OMVs significantly promoted cell migration (Figure 2K–M). Transwell assays further demonstrated that OMVs treatment enhanced both migration and invasion of BCa cells (Figure 2N–Q). Meanwhile, Scratch and transwell experiments of OMVs from *F. n* treated with GW4869 revealed the decline of migration in BCa cells (Figure S2F–J). These findings suggest that *F. nucleatum* exerts its effects on BCa cells largely through OMVs secretion. To further validate the pro‐metastatic role of *F. n–*derived OMVs in vivo, mice bearing popliteal tumors received intratumoral injections of vehicle (PBS) or OMVs. Vivo bioluminescence imaging revealed that OMVs‐treated mice developed lymph node metastasis earlier than control mice (Figure 2R). Accordingly, lymph nodes from the OMVs group were significantly larger than those from the vehicle (PBS) group (Figure 2S). IHC staining for luciferase further confirmed increased tumor cell infiltration in lymph nodes from the OMVs group (Figure 2T,U). Together, these results demonstrate that *F. nucleatum* promotes BCa lymph node metastasis predominantly through the secretion of OMVs, both in vitro and in vivo.

### 2.3 FomA Protein of *F. n* OMVs Promotes BCa Cells Metastasis

**FIGURE 3:**
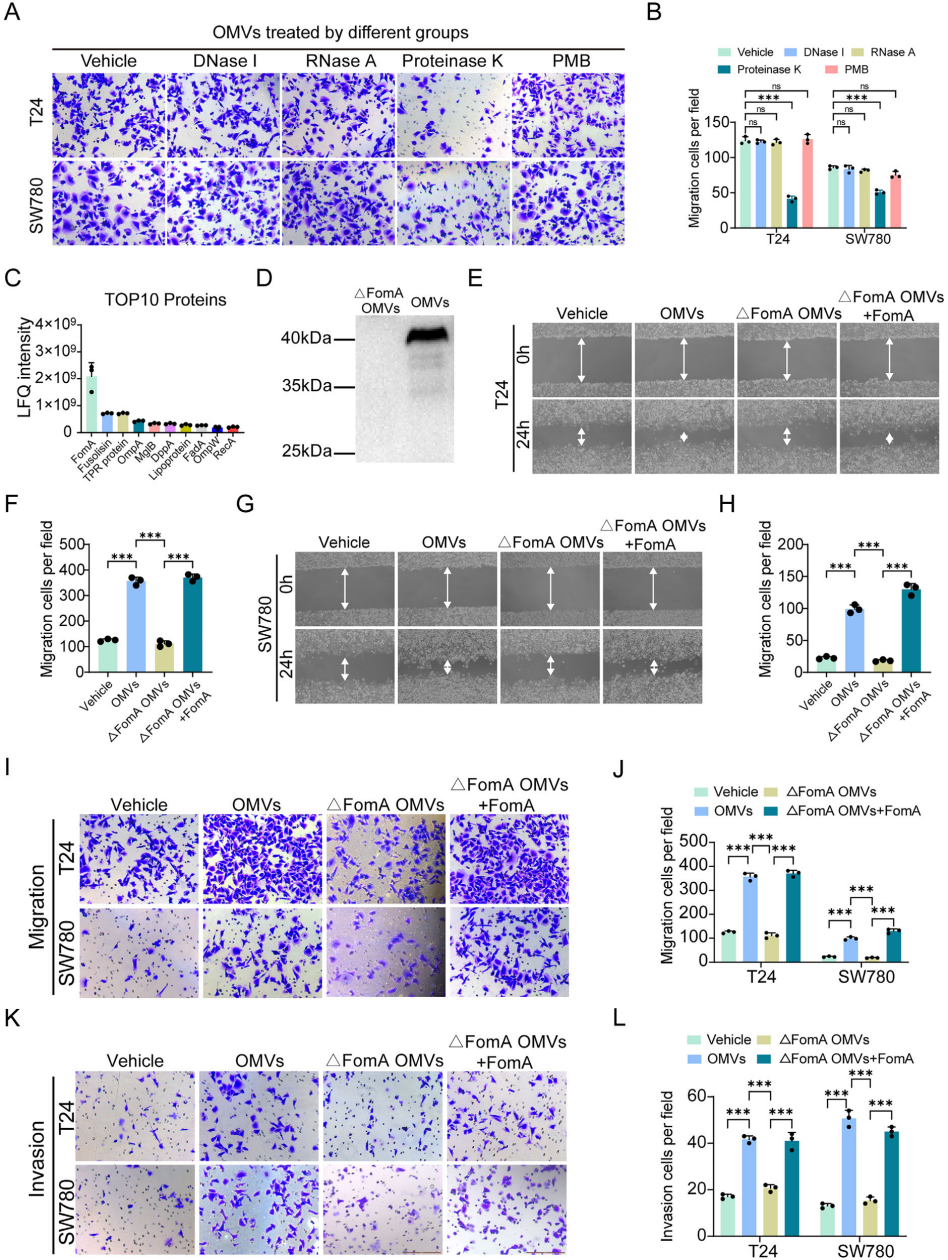
FomA protein of *F. n* OMVs promotes BCa cells metastasis. (A) Representative images showing the effects of OMVs from different treatments on T24 and SW780 cell migration. (B) Quantification of migrated BCa cells. (C) Top 10 proteins with the highest relative expression levels. (D) Western blot analysis of the expression of FomA protein in OMVs. (E–H) Representative images and quantification of scratch assays in BCa cells under different treatments. (I,J) Representative images and quantification of Transwell migration assays in BCa cells under different treatments. (K,L) Representative images and quantification of Transwell invasion assays in BCa cells under different treatments. Data are presented as mean ± SEM, ^*^ *p* < 0.05, ^**^ *p* < 0.01, ^***^ *p* < 0.001. PMB: polymyxin B. ΔFomA OMVs: OMVs from the mutant *F. nucleatum* with depleting FomA through homologous recombination. FomA: *Fusobacterium* outer membrane protein A.

Given the complex composition of OMVs, including lipopolysaccharide (LPS), proteins, and nucleic acids, the specific active ingredients need to be further verified. OMVs were treated with polymyxin B (PMB), DNase I, RNase A, or proteinase K to selectively degrade LPS, DNA, RNA, or proteins, respectively. Transwell assays revealed that OMVs treated with proteinase K did not promote migration significantly (Figure 3A,B), indicating that proteins within OMVs are the primary mediators of their pro‐metastatic effects. To identify key proteins within OMVs from *F. nucleatum* that may contribute to BCa progression, we performed proteomic analysis of OMVs. The FomA protein exhibited the highest relative abundance, consistent with limited literature suggesting a potential biological role of FomA in *F. nucleatum* (Figure 3C). Also, FomA exhibited the strongest ability of pro‐metastasis compared with other proteins. Given its high expression and potential functional importance, FomA was selected for further study. Then, we constructed a mutant *F. nucleatum* with depleting FomA (ΔFomA *F. n*) through homologous recombination and collected the OMVs (ΔFomA OMVs) from this strain. Western blotting analysis confirmed the reduction of FomA protein in OMVs derived from the ΔFomA mutant strain (Figure 3D). To assess the biological impact of FomA, BCa cells were treated with Vehicle (PBS), OMVs, ΔFomA OMVs, and ΔFomA OMVs+FomA. Compared with wild‐type OMVs, ΔFomA OMVs failed to significantly promote BCa cell migration and invasion. Notably, supplementation with FomA restored the pro‐migratory and pro‐invasive effects of ΔFomA OMVs (Figure 3E–L). Collectively, these results demonstrate that FomA is a critical functional component of *F. nucleatum*‐derived OMVs that mediates their tumor‐promoting activity in BCa.

### 2.4 TLR2 as a Downstream Receptor to  FomA is Associated with Poor Prognosis in Bladder Cancer

**FIGURE 4:**
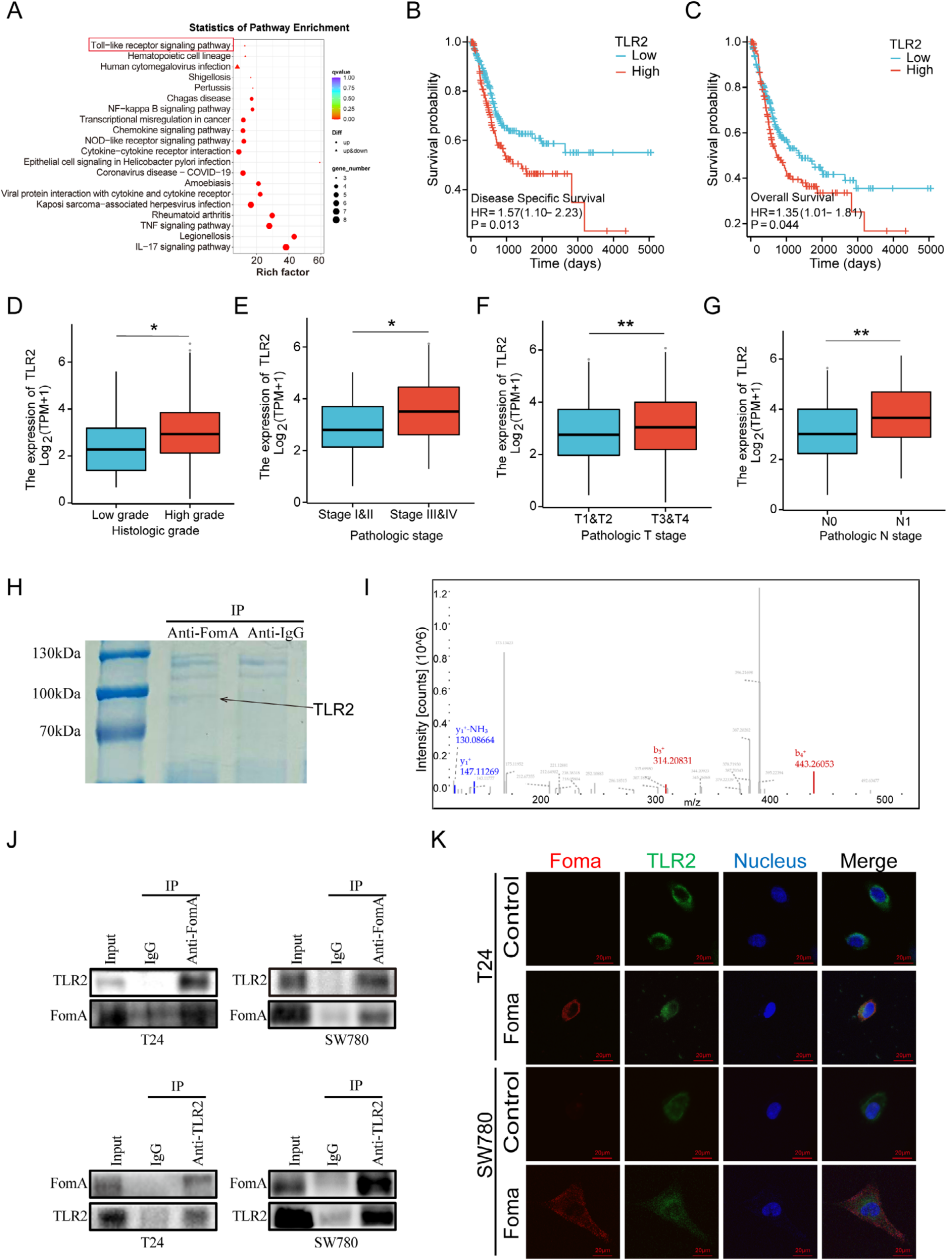
TLR2 as a downstream receptor to FomA is associated with poor prognosis in BCa. (A) KEGG pathway enrichment analysis of differentially expressed genes. (B–G) Association of TLR2 expression with overall survival, disease‐free survival, tumor stage, and tumor grade in BCa patients based on TCGA data. (H) SDS‐PAGE and Coomassie brilliant blue staining. (I) Mass spectrometry analysis of proteins immunoprecipitated with FomA. (J) Co‐immunoprecipitation (Co‐IP) and Western blotting validation of the interaction between FomA and TLR2. (K) Immunofluorescence images showing FomA and TLR2 localization in T24 and SW780 cells under different treatments. Data are presented as mean ± SEM, ^*^ *p* < 0.05, ^**^ *p* < 0.01, ^***^ *p* < 0.001.

To further explore the molecular mechanisms underlying OMVs‐induced BCa progression. RNA‐seq was performed to detect the difference between BCa cells treated with OMVs or PBS. KEGG pathway analysis of RNA‐seq data revealed the enrichment of the Toll‐like receptor signaling pathway following OMVs treatment (Figure 4A). Based on previous articles suggesting an interaction between FomA and TLR2, we analyzed public TCGA datasets to assess the clinical significance of TLR2 expression in BCa. High TLR2 expression was significantly associated with reduced overall survival (OS) and disease‐free survival (DFS), as well as with more advanced tumor grade and stage (Figure 4B–G). To investigate whether FomA interacts with TLR2, we performed immunoprecipitation (IP) experiments. Recombinant FomA protein was able to pull down TLR2, as confirmed by Coomassie Brilliant Blue staining and mass spectrometry analysis (Figure 4H,I). Moreover, co‐immunoprecipitation followed by Western blotting further validated the interaction between FomA and TLR2 in both T24 and SW780 cells (Figure 4J). Consistently, immunofluorescence microscopy demonstrated co‐localization of fluorescently labeled FomA protein (red) and TLR2 (green) on the plasma membrane (Figure 4K). Overall, these results indicate that FomA, a major protein component of *F. nucleatum* OMVs, physically associates with TLR2 and may thereby contribute to lymphatic metastasis in BCa.

### 2.5 FomA Promotes Bladder Cancer Lymphatic Metastasis in a TLR2‐Dependent Manner

**FIGURE 5:**
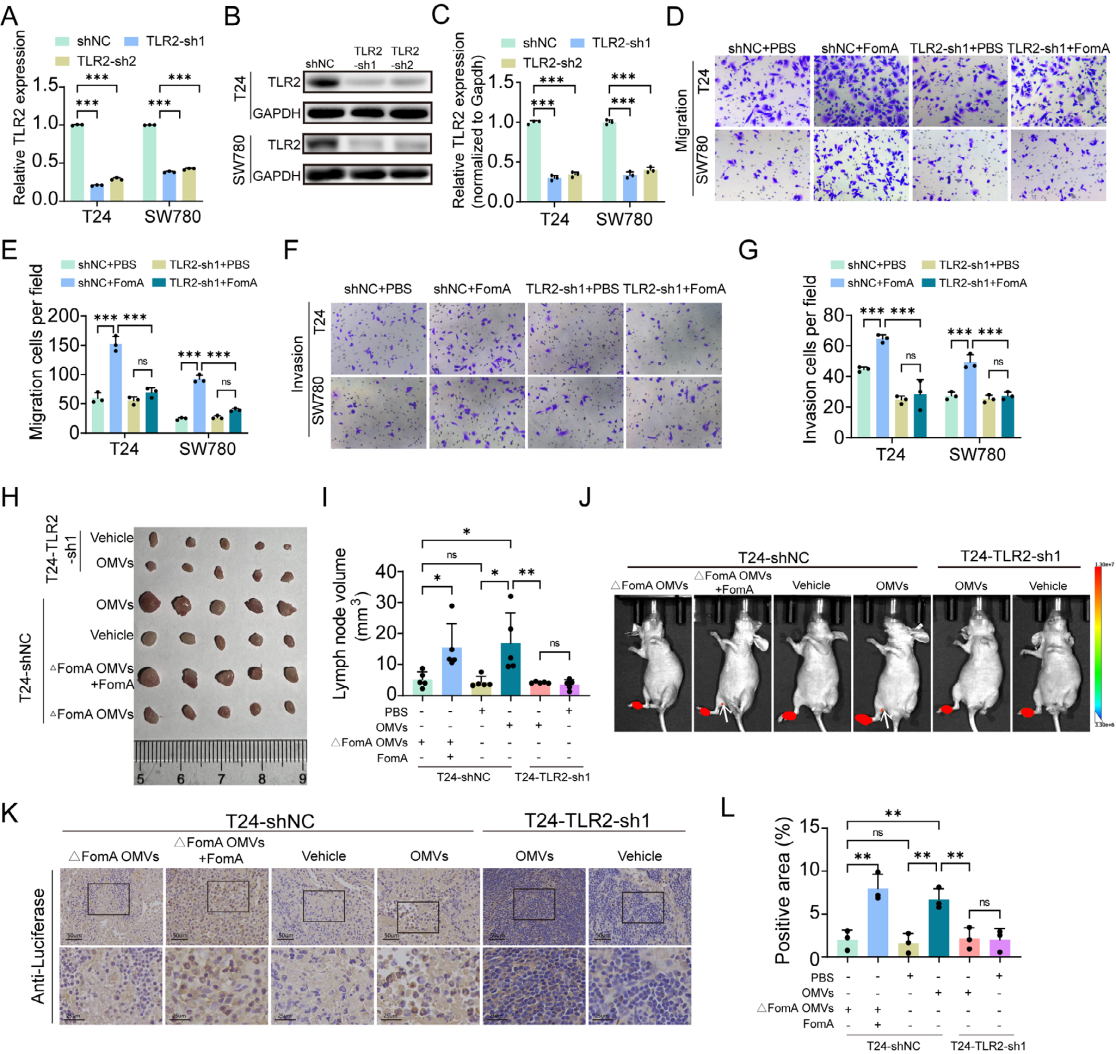
FomA promotes bladder cancer lymphatic metastasis in a TLR2‐dependent manner. (A–C) Relative TLR2 expression was assessed by qPCR (A) and Western blot analysis (B,C) in cells transfected with TLR2‐targeting shRNA or negative control plasmids. (D–G) Representative images of migration, invasion assays, along with quantitative analysis of migrated cells in T24 and SW780 cells under various conditions, including TLR2 knockdown, FomA treatment, and their combination. (H,I) Representative images and quantitative analysis of dissected popliteal LNs (n=5). (J) Representative bioluminescence images. (K,L) Representative IHC staining images. Data are presented as mean±SEM, ^*^ *p* < 0.05, ^**^ *p* < 0.01, ^***^ *p* < 0.001; ns: not significant.

To validate the role of TLR2 in OMVs‐mediated phenotypes, we constructed stable BCa cell lines with TLR2 knockdown using shRNA (TLR2 sh1 and TLR2 sh2) and corresponding negative controls (shNC) (Figure 5A–C). Given its higher knockdown efficiency, TLR2‐sh1 was selected for subsequent experiments. Migration and invasion assays were performed under four conditions: shNC+PBS, shNC+FomA, TLR2 sh1+PBS, and TLR2 sh1+FomA. While FomA treatment significantly enhanced the migratory and invasive capacities of shNC cells, these effects were markedly attenuated in TLR2‐knockdown cells, indicating that TLR2 is required for the pro‐migratory and pro‐invasive effects of FomA derived from *F. nucleatum* OMVs (Figure 5D–G). To further evaluate the role of TLR2 in vivo, luciferase‐expressing T24 BCa cells were used to establish two stable cell lines: T24‐shNC and T24‐TLR2‐sh1. In the T24‐shNC cohort, mice received PBS, OMVs, ΔFomA OMVs, or ΔFomA OMVs supplemented with recombinant FomA, whereas mice in the T24‐TLR2‐sh1 cohort received PBS or OMVs. Notably, in the T24‐shNC group, mice treated with OMVs or △FomA OMVs + FomA resulted in earlier onset and increased burden of lymph node metastasis, as detected by bioluminescence imaging (Figure 5H–J) and further confirmed by luciferase IHC (Figure 5K,L). These results demonstrate that FomA promoted lymph node metastasis. In contrast, TLR2 knockdown suppressed lymph node metastasis, and neither PBS nor OMVs induced detectable metastatic signals in the T24‐TLR2‐sh1 group. Taken together, these findings demonstrate that FomA promotes bladder cancer lymphatic metastasis in a TLR2‐dependent manner, supporting a model in which *F. nucleatum–*derived OMVs drive metastatic progression through activation of the TLR2 signaling pathway.

### 2.6 FomA Engages TLR2 to Activate NF‐κB Signaling and Transcriptional Upregulation of IL‐6

**FIGURE 6:**
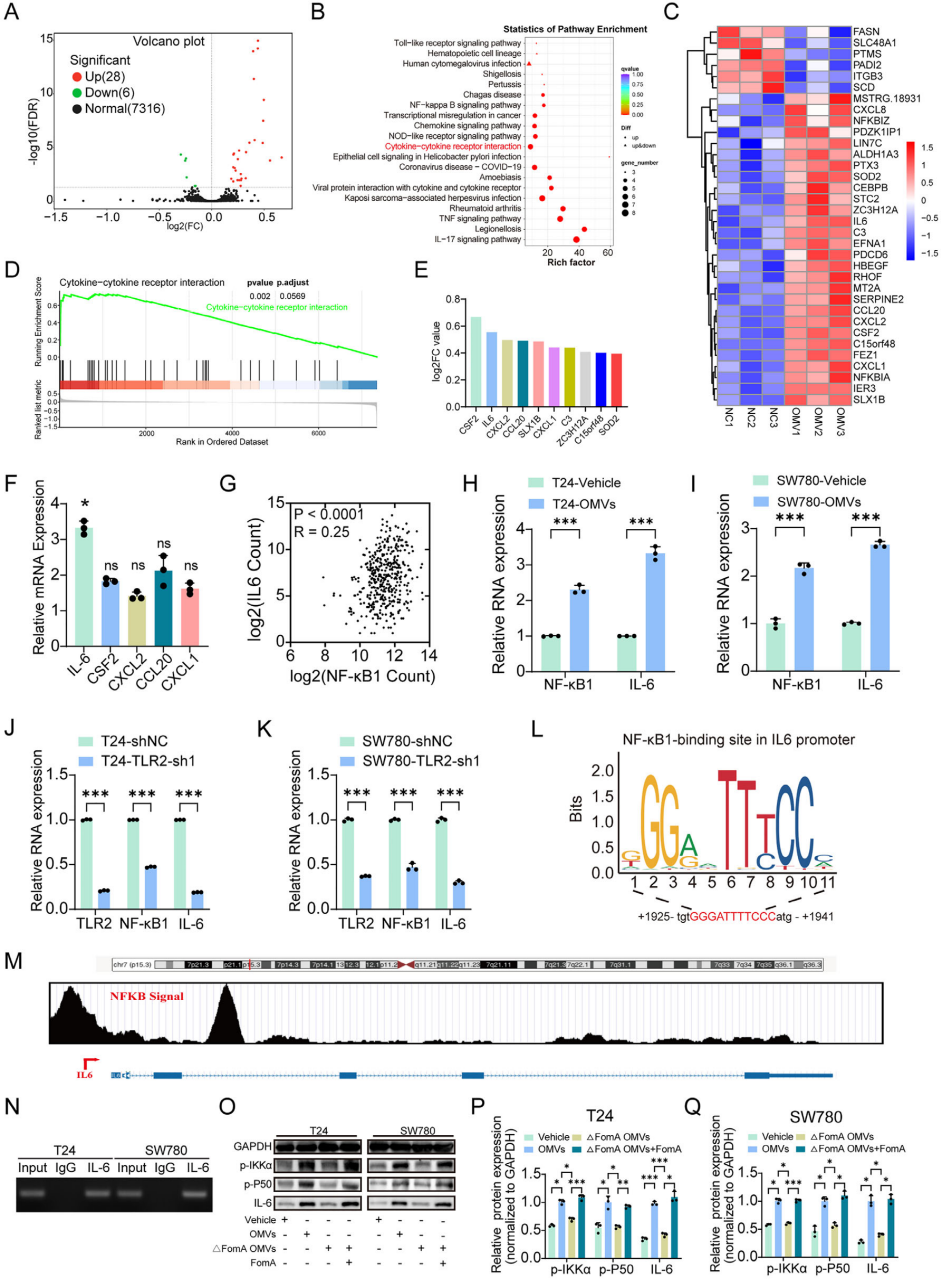
FomA engages TLR2 to activate NF‐κB signaling and transcriptional upregulation of IL‐6. (A) Clustering heatmap of gene expression patterns. (B) Volcano plot of differentially expressed genes. Selection criteria: *p* < 0.05, fold change ≥ 1, FPKM > 5. (C) KEGG pathway enrichment analysis of differentially expressed genes. (D) GSEA enrichment plot. (E) Top 10 differentially expressed genes. (F) The mRNA expression level of different genes. (G) Correlation between NF‐κB1 and IL‐6 expression in BCa based on TCGA data. (H,I) Relative mRNA expression levels of IL‐6 and NF‐κB1 in cells stimulated with OMVs. (J,K) Relative mRNA levels of IL‐6, NF‐κB1, and TLR2 in BCa cells following TLR2 knockdown. (L) Predicted NF‐κB binding motifs in the IL‐6 promoter region based on the JASPAR database. (M) Enrichment of NF‐κB in the IL‐6 promoter region is shown in the UCSC Genome Browser. (N) ChIP‐qPCR and DNA electrophoresis analyses of the binding of NF‐κB to the IL‐6 promoter. (O–Q) Representative Western blot images and quantitative analysis showing expression and phosphorylation of NF‐κB pathway–related proteins under the indicated treatment conditions. Data are presented as mean±SEM, ^*^ *p* < 0.05, ^**^ *p* < 0.01, ^***^ *p* < 0.001; ns: not significant.

The above studies suggest that *F. n* OMVs promote the migration, invasion, and lymphatic metastasis of BCa through FomA in a TLR2‐dependent manner. To further investigate the downstream mechanisms, BCa cells treated with *F. n* OMVs or PBS were subjected to RNA‐seq analysis. After filtering out low‐abundance genes (FPKM < 5), differential expression analysis identified 28 upregulated and 6 downregulated genes, as illustrated by volcano and hierarchical clustering plots (Figure 6A,B). KEGG pathway enrichment analysis of RNA‐seq data revealed that multiple pathways are associated with *F. n* OMVs, with cytokine–cytokine receptor interaction emerging as one of the most significantly enriched pathways (Figure 6C). Consistently, gene set enrichment analysis (GSEA) further confirmed prominent enrichment of cytokine‐related signaling pathways in OMVs‐treated cells (Figure 6D). Among the top 10 differentially expressed genes, the majority were cytokines (Figure 6E). To validate these findings, we performed quantitative PCR analysis targeting a panel of cytokines, including IL6, CSF2, CXCL1, CXCL2, and CCL20. IL6 exhibited the most upregulation in response to OMV stimulation (Figure 6F). Given that IL‐6 is a well‐established downstream effector of Toll‐like receptor signaling, we next assessed the clinical relevance of IL‐6 in Bca. Analysis of TCGA‐BLCA datasets revealed that high IL‐6 expression was significantly associated with reduced overall survival (OS) and disease‐free survival (DFS). Moreover, elevated IL6 expression correlated positively with higher tumor grade and more advanced T and N stages (Figure S3A–F). Our previous experiments demonstrated that TLR2 and *F. nucleatum* outer membrane vesicles (OMVs) are functionally involved in promoting BCa progression. Activation of TLR2 induces the canonical NF‐κB signaling cascade, a well‐characterized pathway in cancer known for its transcriptional regulatory role. Consistent with this, KEGG analysis of RNA‐seq data revealed significant enrichment of the NF‐κB signaling pathway following OMVs treatment (Figure S3G). To investigate whether NF‐κB acts as a molecular link between TLR2 activation and IL‐6 upregulation, we examined the relationship between NF‐κB1 and IL‐6 expression. Pearson correlation analysis of TCGA‐BLCA data demonstrated a strong positive correlation between NF‐κB1 (also known as NF‐κB) and IL6 transcript levels (Figure 6G). In vitro, OMVs stimulation of T24 cells led to increased expression of both NF‐κB1 and IL‐6 (Figure 6H,I). This induction was significantly attenuated in TLR2‐knockdown cells, as confirmed by qPCR (Figure 6J,K), suggesting that OMV‐induced activation of NF‐κB1 and IL‐6 is dependent on TLR2 signaling.

To further investigate the transcriptional regulation of IL‐6 by NF‐κB1, we analyzed the promoter region of IL6. JASPAR prediction identified NF‐κB1 binding sites within the 2000 bp upstream region of the IL‐6 promoter (Figure 6L). Consistently, Cistrome DB predictions revealed a strong NF‐κB1 binding peak within the IL6 promoter region (Figure 6M). Chromatin immunoprecipitation (ChIP) assays validated the direct binding of NF‐κB1 to the IL6 promoter, confirming its transcriptional regulatory role (Figure 6N). Furthermore, western blot analysis showed that exposure to OMVs or FomA resulted in increased levels of phosphorylated IKK‐α, phosphorylated p50, and IL‐6 (Figure 6O–Q). Collectively, these findings reveal that *F. n* OMVs activate the TLR2/NF‐κB/IL‐6 signaling axis in BCa cells.

### 2.7 FomA‐Containing OMVs From *F. n* Facilitate Lymph Node Metastasis Through IL‐6 Dependent M2b Macrophage Recruitment

In preliminary in vitro assays, OMVs exerted minimal effects on BCa cell proliferation (Figure S5A,B). However, in vivo experiments revealed that OMVs treatment significantly promoted tumor growth, as evidenced by increased tumor volume (Figure S5C,D), suggesting that OMVs may enhance tumor progression through modulation of the tumor microenvironment rather than direct effects on tumor cell proliferation. Consistently, analysis of public datasets revealed a significant positive correlation between IL‐6/TLR2 signaling and macrophage infiltration in BCa tissues (Figure S5E–H). Based on these findings, we hypothesized that FomA‐containing OMVs promote BCa progression by regulating macrophage recruitment and polarization.

**FIGURE 7:**
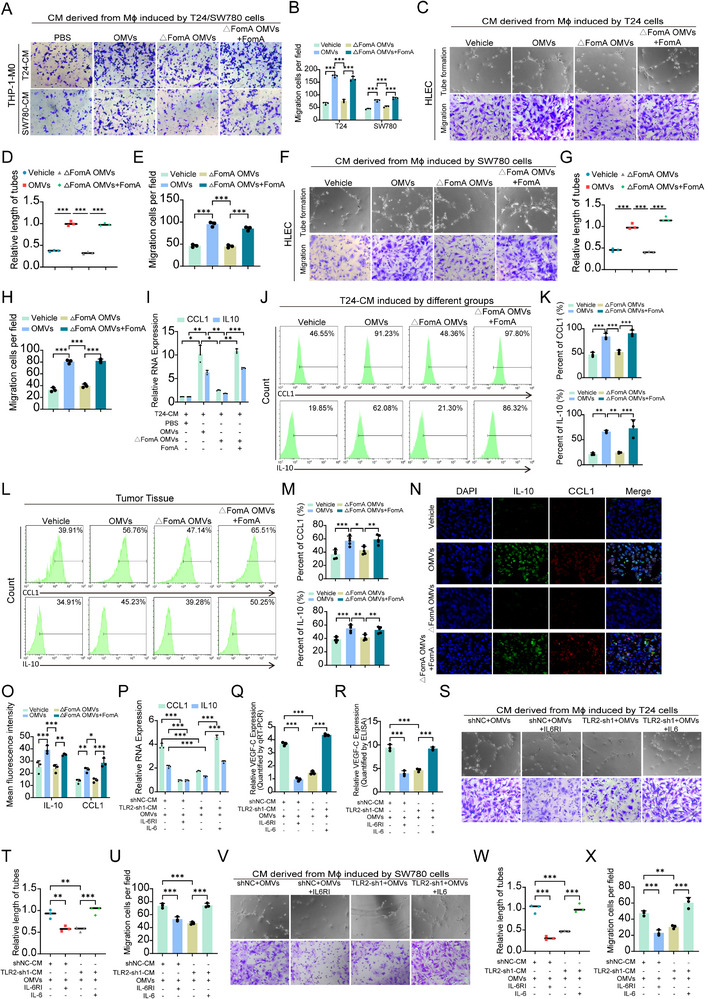
FomA‐containing OMVs from *F. n* facilitate lymph node metastasis through IL‐6‐dependent M2b macrophage recruitment. (A,B) Representative images and quantification of THP‐1‐derived macrophage (THP‐1‐Mϕ) migration induced by conditioned media (CM) from BCa cells subjected to the indicated treatments. (C–H) Representative images and quantitative analysis of HLEC tube formation and migration induced by CM from T24 (C–E) and SW780 (F–H) cells under different treatments. (I) Relative mRNA expression levels of the M2b‐associated markers CCL1 and IL‐10. (J,K) Flow cytometry analysis of CCL1 and IL‐10 expression in macrophages treated with supernatants from differently treated T24 cells. (L,M) Flow cytometry detection of CCL1 and IL‐10 expression in tumor‐infiltrating macrophages from tumor tissues of mice. (N,O) Representative immunofluorescence images and quantitative analysis of CCL1 and IL‐10 expression in tumor sections. (P) Relative mRNA expression of CCL1 and IL‐10 in macrophages, treated with T24‐CM, T24 TLR2‐sh1‐CM, IL‐6, and IL‐6RI under OMVs stimulation. (Q,R) Quantitative PCR and ELISA analysis of CCL1 and IL‐10 expression in macrophages treated with CM from T24‐CM, T24 TLR2‐sh1‐CM, IL‐6, and IL‐6RI under OMVs stimulation. (S–X) Representative images and quantitative analysis of HLEC tube formation and migration induced by CM from T24 (S‐U) and SW780 (V‐X) cells under different treatments. Data are presented as mean±SEM, ^*^ *p* < 0.05, ^**^ *p* < 0.01, ^***^ *p* < 0.001; ns: not significant. CM: conditional medium. IL‐6RI: IL‐6 receptor inhibitor.

BCa cells were treated with PBS, OMVs, △FomA OMVs, or △FomA OMVs+FomA, and the supernatants were collected as conditioned media (CM). Human THP‐1 monocytes were differentiated into macrophage‐like cells using PMA (100 ng/mL) and exposed to the CM. Transwell migration assays demonstrated that macrophages treated with CM derived from OMVs or △FomA OMVs+FomA exhibited significantly enhanced migration compared to those treated with PBS or △FomA OMVs CM (Figure 7A,B). Additionally, CM from these macrophages promoted the migration of BCa cells (Figure S4A,B). In parallel, OMVs and FomA‐supplemented OMVs enhanced tube formation and migration of human lymphatic endothelial cells (HLECs), indicating a potential pro‐lymphangiogenic effect (Figure 7C–H).

Previous studies have indicated that M2b macrophage polarization is typically driven by immune complexes or TLR agonists. Given the role of TLR2 in *F. nucleatum*‐mediated BCa progression, we examined macrophage polarization by exposing M0 macrophages to CM from the indicated treatment groups. Quantitative PCR analysis showed that expression levels of CCL1 and IL‐10 were significantly increased in macrophages treated with OMVs or ΔFomA OMVs supplemented with FomA (Figure 7I). These results were further validated by ELISA (Figure S5I–L) and flow cytometry (Figure 7J,K). In tumor tissues from the mouse model, CCL1 and IL‐10 expression were significantly elevated in the OMVs and △FomA OMVs+FomA groups compared with controls (Figure 7L,M). Immunofluorescence staining further confirmed increased CCL1 and IL‐10 expression within tumor sections from these groups (Figure 7N‐O).

In addition, to investigate the roles of IL‐6 and TLR2 in macrophage polarization, macrophages were treated with CM derived from the following groups: shNC+OMVs, shNC+OMVs+IL‐6 receptor inhibitor (IL‐6RI), shTLR2+OMVs, and shTLR2+OMVs+recombinant IL‐6. PCR analysis showed that inhibition of IL‐6 signaling or TLR2 knockdown significantly reduced CCL1 and IL‐10 expression, whereas supplementation with IL‐6 restored their expression to baseline levels (Figure 7P). In line with these findings, increased secretion of CCL1, IL‐6, and IL‐10 by polarized macrophages further facilitated BCa cells migration (Figure S4C,D). Because tumor‐associated macrophage polarization has been linked to lymphangiogenesis, we next examined the expression of VEGF‐C, a key regulator of lymphatic vessel formation and lymph node metastasis. ELISA and qPCR analyses revealed that TLR2 knockdown or inhibition of IL‐6 signaling significantly reduced VEGF‐C expression, whereas IL‐6 supplementation restored VEGF‐C levels (Figure 7Q,R). Consistently, tube formation and migration assays using T24 and SW780 cells further supported the pro‐lymphangiogenic effects mediated by this pathway (Figure 7S‐X). Collectively, these results demonstrate that FomA‐containing OMVs from *F. nucleatum* remodel the tumor microenvironment by inducing IL‐6–dependent polarization of macrophages toward an M2b phenotype, thereby enhancing VEGF‐C expression and lymph node metastasis in BCa.

### 2.8 Pinocembrin Attenuates FomA‐Mediated Bladder Cancer Progression by Targeting the FomA–TLR2 Interaction

**FIGURE 8:**
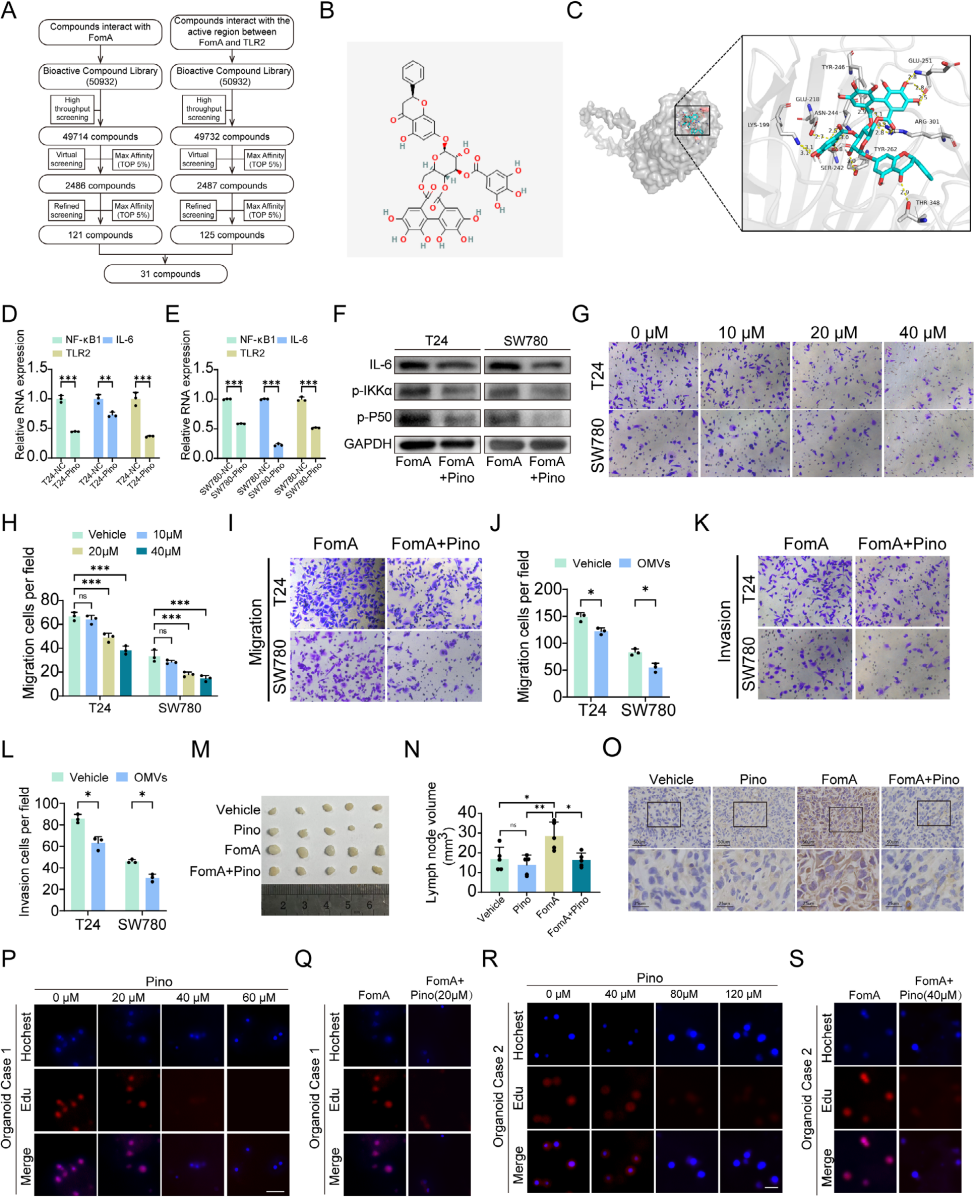
Pinocembrin attenuates FomA‐mediated bladder cancer progression by targeting the FomA–TLR2 interaction. (A) Workflow of structure‐based virtual screening of small‐molecule compounds targeting the predicted FomA–TLR2 interaction interface. (B) Chemical structure of pinocembrin. (C) Autodock Vina of pinocembrin into the active pocket of the FomA protein. (D–F) Quantitative PCR analysis and western blot analysis of TLR2, NF‐κB1, and IL‐6 mRNA and protein expression in T24 and SW780 cells. (F) Western blot analysis of NF‐κB signaling‐related protein expression in T24 and SW780 cells treated with FomA or FomA+Pinocembrin. (G,H) Migration assays showing the effects of increasing concentrations of pinocembrin on the migratory capacity of T24 and SW780 cells. (I–L) Representative images and quantitative analysis of migration and invasion assays in T24 and SW780 cells treated with FomA alone or in combination with pinocembrin (10 µm). (M) Representative image of dissected popliteal LNs (n=5). (N) Quantitative analysis of LNs volumes. (O) Representative images of IHC staining. (P–S) Proliferation analysis of patient‐derived bladder cancer organoids (two cases with LN metastasis) treated with increasing concentrations of pinocembrin or with FomA alone or in combination with pinocembrin. Data are presented as mean±SEM, ^*^ *p* < 0.05, ^**^ *p* < 0.01, ^***^ *p* < 0.001; ns: not significant. IC50: half‐maximal inhibitory concentration. Pino: pinocembrin.

To further investigate the therapeutic potential of FomA inhibition in BCa, we performed an in silico screening to identify small‐molecule inhibitors capable of interfering with the FomA–TLR2 interaction. Based on docking analyses targeting the predicted interaction interface between FomA and TLR2, a total of 31 candidate compounds were initially screened from multiple compound libraries (Figure 8A). Using AutoDock Vina, five compounds with the highest docking scores were selected for further experimental validation (Table S3). Among these candidates, pinocembrin (pino) exhibited the strongest inhibitory effect on FomA‐associated signaling. The chemical structure of pinocembrin and the predicted binding region between FomA and TLR2 at the FomA–TLR2 interaction interface were presented (Figure 8B,C). The half‐maximal inhibitory concentrations (IC_50_) of the candidate compounds were determined (Figure S7A,B), and concentrations that did not significantly affect BCa cell viability were selected for subsequent experiments. Quantitative PCR and Western blot analysis demonstrated that pinocembrin treatment significantly reduced the expression of TLR2, NF‐κB1, and IL‐6 in BCa cells (Figure 8D–F; Figure S8A,B). We next evaluated the effects of pinocembrin on BCa cell migratory capacity. A pronounced inhibitory effect was observed at a concentration of 20 µm, whereas 10 µm exerted minimal effects on cell migration (Figure 8G,H). Importantly, low‐dose pinocembrin suppressed BCa cell growth while exerting minimal effects on immortalized human bladder epithelial cells (SV‐HUC‐1) (Figure S7C,D). Based on these findings, 10 µm pinocembrin was selected for subsequent combination experiments with recombinant FomA. Co‐treatment with pinocembrin significantly attenuated FomA‐induced enhancement of BCa cell migration in vitro (Figure 8I–L). Consistently, in vivo experiments demonstrated that pinocembrin administration markedly reduced the ability of FomA to promote lymph node metastasis (Figure 8M–O; Figure S8C). To further assess the translational relevance of these findings, patient‐derived organoids were established from tumor tissues of two BCa patients with lymph node metastasis. EdU incorporation assays were performed to determine an appropriate concentration of pinocembrin, and the results showed that pinocembrin significantly attenuated the pro‐metastatic effects of FomA in BCa organoids (Figure 8P–S; Figure S8D–G). Notably, intratumoral administration of pinocembrin did not induce overt toxicity or adverse effects in treated mice, as assessed by body weight, histological analysis, and serum biochemical parameters (Figure S6A–G). Collectively, these results demonstrate that pinocembrin effectively suppresses BCa cell migration and metastatic progression by targeting the FomA–TLR2 interaction, highlighting its potential as a therapeutic agent for BCa.

## 3 Discussion

Bladder cancer (BCa) is continuously exposed to the urinary microbiota, yet the biological significance of this interaction in tumor progression has remained largely unexplored. In this study, we provide comprehensive evidence that *F. nucleatum* represents a key microbial driver of lymph node (LN) metastasis in BCa. By integrating urinary microbiome profiling, tumor‐resident microbial analysis, functional assays, and molecular mechanistic studies, we demonstrate that *F. nucleatum* is selectively enriched in LN‐positive BCa and actively promotes tumor dissemination through the release of outer membrane vesicles (OMVs). These vesicles are highly enriched in the outer membrane protein FomA, which functions as a microbial signaling ligand that engages TLR2 on BCa cells, activates NF‐κB‐dependent IL‐6 transcription, and integrates both tumor‐intrinsic invasiveness and tumor microenvironment remodeling.

The bladder occupies a unique anatomical and physiological niche as a urine storage organ that is continuously bathed in microbial products. Although urine was long considered sterile, advances in sequencing technologies have revealed a diverse urinary microbiome even in healthy individuals [6]. BCa further disrupts this microbial ecosystem [33, 34], yet the functional consequences of this dysbiosis have been poorly understood. Most studies of BCa metastasis have focused on tumor cell‐intrinsic alterations or stromal interactions, leaving the potential contribution of microbial communities to LN dissemination largely unexplored. Lymph node metastasis is the primary route of dissemination in BCa and a key factor contributing to poor prognosis [35]. Given that LN metastasis is the predominant route of systemic spread in BCa and a major determinant of patient prognosis, understanding whether microbial signals participate in this process represents a critical conceptual gap. In this study, by performing 16S rDNA sequencing of urine from BCa patients and healthy controls, we identified *Fusobacterium* as one of the most significantly enriched genera in BCa, with a particularly strong association with LN‐positive disease. This observation was reinforced by tumor‐resident microbial profiling and qPCR analysis, which demonstrated that *F. nucleatum* is markedly more abundant in tumor tissues from LN‐positive patients compared with LN‐negative tumors and normal adjacent tissues. These findings suggest that *F. nucleatum* is not merely a bystander but may function as a metastasis‐associated pathobiont in BCa. This concept is consistent with studies in various types of tumors. Chen et al. found that *F. nucleatum* promotes colorectal cancer metastasis through the miR‐1322/CCL20 axis and M2 polarization [28]. Zhuo et al. found that *F. nucleatum* reduces METTL3‐mediated m6A modification and leads to colorectal cancer metastasis [36]. In addition, tumor‐associated microbiota is becoming popular as a way to explore another avenue for exploring novel bacterial anticancer therapies. Cai et al. found that tumor‐resident microbiota promoted metastatic colonization of breast cancer [37]. Straussman et al. found that intratumoral bacteria play an important role in mediating colorectal rectal tumors' resistance to gemcitabine chemotherapeutic agents [38].

An important insight from our study is that *F. nucleatum* exerts its oncogenic effects primarily through OMVs rather than through direct bacterial colonization. OMVs represent nanoscale lipid bilayer vesicles that encapsulate proteins, nucleic acids, and pathogen‐associated molecular patterns, enabling bacteria to communicate with host cells over distance. We found that pharmacological inhibition of vesicle release by GW4869 significantly reduced BCa cell migration and invasion, while protease K treatment markedly attenuated the pro‐tumorigenic activity of OMVs, indicating that protein cargoes within OMVs are critical mediators of tumor progression. These findings place *F. nucleatum*‐derived OMVs within a growing class of microbial vesicles that function as active regulators of tumor biology. Similarly, OMVs of microbiota can promote metastasis of oral cancer [39], as well as Qu et al. found that the antibiotic metronidazole could be packed in a metal frame containing Fe and then encapsulated in *F. n* OMVs, thus targeting and eliminating bacterial impact on tumor cells by nanomaterial technology [40]. Recent studies have shown that bacterial outer membrane vesicles (OMVs) engineered using CRISPR‐based systems can potentiate antitumor T cell immunity by facilitating the delivery of tumor antigens and pathogen‐associated molecular patterns to antigen‐presenting cells [41]. Bacterial OMVs can function as endogenous nanovaccines that deliver pathogen‐associated molecular patterns and tumor antigens to antigen‐presenting cells, thereby activating both innate and adaptive immunity. Preclinical studies have shown that engineered OMVs reprogram tumor‐associated macrophages, enhance dendritic cell maturation, and synergize with immune checkpoint blockade to suppress tumor growth and metastasis [42, 43, 44]. Our findings extend this paradigm by showing that pathogenic OMVs derived from *F. nucleatum* can actively drive BCa progression, highlighting the dual nature of bacterial vesicles as both potential therapeutic tools and endogenous tumor‐promoting factors.

Therefore, further exploration of bacteria and incorporation of OMVs can directly explore the mechanism of microorganisms in the progression of bladder tumors. The rich material components within the outer membrane vesicles. Including nucleic acids, peptidoglycan, lipopolysaccharides, outer membrane proteins, etc [45]. Among OMVs with different treatments, the protease K group reduced the cell migration ability. The result showed that the protein in OMVs may play a key role in the progression of BCa. Previous articles have revealed that bacterial outer membrane vesicles have a variety of important biological functions. In this study, proteinase K treatment diminished the protumor effect of OMVs, highlighting the biological function of OMVs and their proteins [46]. Within *F. nucleatum* OMVs, we identify FomA as the principal pro‐tumorigenic effector.

FomA, a porin family protein, is known to mediate bacterial adhesion and invasion, biofilm formation, and immune recognition. Previous studies implicated FomA in colorectal cancer and periodontal disease via interactions with other pathogens [16, 47]. Despite this, its role in BCa remained unexplored. OMVs derived from FomA‐deletion strains (△FomA) showed reduced tumor‐promoting effects, which were restored upon exogenous FomA complementation, revealing the essential role of FomA in OMV‐mediated oncogenicity. △FomA *F. n* also indicated the importance of the construction of engineered bacteria in tumor development. Here, we demonstrate that FomA is highly enriched in OMVs. Notably, FomA may act as a TLR2 agonist, consistent with findings from gut epithelial studies, and is sufficient to activate classical NF‐κB signaling, leading to IL‐6 transcription [19, 48]. Through RNA‐seq and qPCR analysis, we found that FomA activated the classical NF‐κB signaling pathway and upregulated the expression of IL‐6 by binding to the TLR2 receptor. IL‐6 is an important cytokine that plays a key role in the tumor microenvironment, especially in promoting tumor invasion and metastasis [49, 50]. Through Western blotting and ChIP experiments, we confirmed that *F. n* OMVs activate TLR2, leading to NF‐κB activation and enhanced binding of NF‐κB to the IL‐6 promoter, thereby promoting IL‐6 transcription and expression. Although the precise binding interface between FomA and TLR2 remains to be defined, FomA is an outer‐membrane β‐barrel protein with multiple surface‐exposed extracellular loops, which are well positioned to function as pathogen‐associated molecular patterns for TLR2 recognition [51, 52]. Together, these data identify FomA as a surface‐exposed microbial factor that drives pro‐tumorigenic signaling in bladder cancer, supporting its potential as a therapeutic target. In our study, the FomA protein plays a promoting role in the progression of BCa through the TLR2/NF‐κB/IL‐6 signaling pathway, which provides theoretical support for FomA as a new target for targeted therapy of BCa. However, IL‐6 not only directly promotes the migration of tumor cells but also can promote the polarization of M2b macrophage [53, 54]. Tumor‐associated macrophages (TAMs), particularly M2‐polarized subsets, are well‐established drivers of LN metastasis in BCa through their secretion of VEGF‐C. Clinically, high TAM infiltration strongly correlates with LN‐positive disease, advanced stage, and poor prognosis [55, 56, 57].

In our study, conditioned media (CM) from BCa cells treated with *F. n* OMVs induced polarization of macrophages toward the M2b phenotype, characterized by elevated CCL1 and IL‐10 expression. The same trend of the expression and secretion of VEGF‐C was also observed. Further, TLR2 knockdown or IL‐6 receptor blockade reduced the ability of CM to enhance macrophage migration and lymphangiogenesis, indicating a key role for IL‐6 downstream under TLR2 activation. IL‐6, a key inflammatory mediator downstream of TLR2/NF‐κB signaling, not only directly enhances tumor cell migration but also actively remodels the tumor microenvironment by promoting macrophage polarization toward the M2b phenotype [54]. Our data show that FomA‐induced IL‐6 promotes M2b polarization and enhances VEGF‐C production. As a master regulator of lymphangiogenesis, VEGF‐C facilitates tumor cell entry into lymphatic vessels, thereby creating a microenvironment that favors lymphatic dissemination [27].

Finally, the identification of FomA as a druggable microbial effector provides a new therapeutic opportunity. Through structure‐guided small‐molecule screening, we identified pinocembrin as a compound that preferentially binds a predicted surface pocket on FomA overlapping the TLR2‐interacting region. Pinocembrin treatment attenuated FomA‐induced NF‐κB activation, IL‐6 expression, macrophage polarization, and tumor progression, supporting its functional role as an inhibitor of the FomA–TLR2 axis. Previous literature has also reported that Pinocembrin can inhibit other tumor progression [58, 59]. Although indirect effects on downstream signaling cannot be excluded, these findings highlight the feasibility of pharmacologically disrupting microbe–tumor communication.

In summary, our study suggests that urinary or intratumoral detection of *F. nucleatum* or FomA may serve as a biomarker for LN metastasis risk in BCa. From a therapeutic perspective, targeting microbial outer membrane vesicles (OMVs) or their key effectors may represent a promising strategy to complement existing immunotherapies. Nevertheless, several limitations should be acknowledged. First, this study was conducted at a single center with a relatively limited sample size, which may restrict the generalizability of our findings. Validation in larger, multi‐center clinical cohorts will be essential for future clinical translation. Second, interactions among microbial communities were not systematically explored. Given the complexity of the tumor‐associated microbiome, future studies should consider interspecies interactions and their collective impact on metastatic progression. Third, although our data support a functional link between *F. nucleatum* and macrophage‐mediated lymphatic metastasis, the precise molecular mechanisms underlying this interaction warrant further investigation. In addition, *F. nucleatum* is an anaerobic bacterium commonly residing in the oral cavity, gastrointestinal tract, and other mucosal sites. It is therefore plausible that OMVs derived from these distant anatomical locations may influence tumor progression either in concert with, or independently of, the bladder‐local microbiome. Elucidating the relative contributions of local versus systemic microbial OMVs represents an important direction for future research. Despite these limitations, our findings identify tumor‐associated microbiota as active regulators of lymphatic metastasis in BCa, thereby expanding current paradigms of metastatic regulation. Collectively, this study highlights the diagnostic potential of urinary or tissue‐based detection of *F. nucleatum* or FomA and provides a conceptual framework for targeting tumor‐associated microbiota as a therapeutic strategy to restrain lymphatic dissemination in BCa.

## 4 Methods

### 4.1 Patient Cohort and Sample Collection

All bladder cancer cases were histopathologically confirmed as urothelial carcinoma based on postoperative paraffin‐embedded tissue analysis. Patients diagnosed with carcinoma in situ were excluded following independent assessment by two senior pathologists. In addition, none of the enrolled patients had received neoadjuvant chemotherapy, radiotherapy, immunotherapy, or other antitumor treatments prior to sample collection. Participants, including patients with bladder cancer and healthy control subjects, were excluded if they met any of the following criteria: (1) exposure to antibiotics, proton pump inhibitors, or other medications known to significantly influence the urinary microbiota within one month before urine collection; or (2) a documented history of urinary tract–related inflammatory or infectious conditions that could affect microbial homeostasis. Urine samples were obtained from all participants during spontaneous voiding using sterile containers and were immediately processed and stored at −80 °C within 2 h of collection. This study was reviewed and approved by the Ethics Committee of Southern Medical University (approval number: NFEC‐2020‐123), and written informed consent was obtained from all participants prior to enrollment.

### 4.2 Animal Models, Cell Lines and Cell Culture

Animals were acquired from SPF Biotechnology Co., Ltd in Beijing, China, and maintained under specific pathogen‐free (SPF) conditions. All the mice were randomly assigned to experimental or control groups to reduce selection bias. T24 cells (2 × 106 cells) resuspended in 200 µL of RPMI 1640 without FBS were injected subcutaneously into the right flank of the mice on day 0. Intratumoral injections were administered three times a week until the mice were sacrificed. Tumors were harvested postmortem for subsequent experiments and further analysis. For the popliteal lymph node metastasis model, BALB/c nude mice were injected with T24 (2 × 106 cells) resuspended in 30 µL of serum‐free 1640 medium into their footpads. The condition of the popliteal LNs was subsequently monitored and imaged with a Vivo Imaging System (N28750). The animal experiments were approved by the Animal Ethics and Welfare Committee, Nanfang Hospital, Southern Medical University, and were performed following the institutional guidelines. Human bladder cancer cell lines T24 (RRID: CVCL_0554), SW780 (RRID: CVCL_5G49), and the immortalized urothelial cell line SV‐HUC‐1 (RRID: CVCL_3798) were obtained from the American Type Culture Collection (ATCC, Manassas, VA, USA) between 2021 and 2024. Human lymphatic endothelial cells (HLECs, Cat. No. 2500, RRID: CVCL_1H44) were purchased from ScienCell Research Laboratories (Carlsbad, CA, USA) in 2023. All cells were maintained at 37°C in a humidified incubator with 5% CO_2_. Culture conditions: SW780 cells were cultured in DMEM medium (Gibco, ThermoFisher Scientific) supplemented with 10% fetal bovine serum (FBS) and 1% penicillin‐streptomycin. T24 cells were maintained in RPMI‐1640 medium (Gibco, ThermoFisher Scientific) with 10% FBS and 1% penicillin‐streptomycin. SV‐HUC‐1 cells were cultured in F‐12K medium (Hyclone) supplemented with 10% FBS and 1% penicillin‐streptomycin. HLECs were cultured in endothelial cell medium (ECM, ScienCell) supplemented with 5% FBS, according to the supplier's protocol. All cell lines were authenticated using short tandem repeat (STR) analysis before experimental use and were confirmed to be free of mycoplasma contamination by PCR‐based testing.

### 4.3 Isolation of Bacterial Outer Membrane Vesicles (OMVs)

Bacterial cultures were grown to the logarithmic phase and centrifuged at 10,000 × g for 30 min at 4 ℃ to remove bacterial cells. The supernatant was filtered through a 0.22‐µm membrane and subjected to ultracentrifugation at 160,000 × g for 2 h at 4 ℃ to pellet outer membrane vesicles (OMVs). The OMV pellet was washed three times with sterile PBS by repeated ultracentrifugation under the same conditions to remove contaminants. Purified OMVs were resuspended in PBS, aliquoted, and stored at −80 ℃ until use. OMVs were characterized by transmission electron microscopy (TEM) and nanoparticle tracking analysis (NTA), and enrichment of OMV‐associated protein markers was verified by Western blotting.

### 4.4 qPCR/ Real‐Time Quantitative PCR

Total RNA was isolated, and quantitative real‐time PCR (qPCR) assays were executed based on established protocols. RNA extraction was performed with TRIzol reagent obtained from Pricella Biotechnology (Wuhan, China). Subsequently, complementary DNA (cDNA) was synthesized using HiScript III RT SuperMix (Vazyme Biotech, Nanjing, China). Expression levels of target RNAs across multiple cell lines were quantified by RT‐qPCR. Primer information utilized in the current experiments can be found in Supplementary Table S1.

### 4.5 Cell Proliferation Assay

Cells (T24, SW780, and SVHUC‐1) were trypsinized and plated into 96‐well plates at 2000 cells/well. Following the indicated treatments, cell viability was assessed by adding CCK‐8 solution following the supplier's protocol. Absorbance was recorded at a wavelength of 450 nm.

### 4.6 Wound Healing Assay

T24 and SW780 cells were seeded in 6‐well plates at a density of 500 000 cells per well in 3 mL of RPMI 1640 or DMEM medium. Once cells reached ∼90% confluence, straight scratches were made in the cell layer. After washing with PBS, the cells were treated, and wound healing was monitored and photographed using a light microscope.

### 4.7 Migration and Invasion Assays

Cell migration and invasion abilities were evaluated using Transwell chambers (8 µm pore size). For migration assays, T24 and SW780 cells (2 × 10⁴ cells) were suspended in 200 µL serum‐free RPMI 1640 or DMEM medium and seeded into the upper chamber. The lower chamber was filled with 600 µL corresponding medium supplemented with 10% fetal bovine serum containing control or experimental treatments. For invasion assays, Transwell inserts were pre‐coated with Matrigel (Corning). Briefly, Matrigel was thawed overnight at 4 °C. Pipette tips and microcentrifuge tubes were pre‐cooled to prevent premature gelation. Under cold conditions, Matrigel was diluted 1:8 (v/v) with serum‐free culture medium and added to the upper surface of each insert, followed by incubation at 37 °C to allow polymerization and formation of a uniform gel layer. Cells were then seeded onto Matrigel‐coated inserts following the same procedure as described for migration assays. After incubation at 37 °C, migrated cells were allowed to pass through the membrane for 4 h, whereas invaded cells were incubated for 24 h in a humidified incubator with 5% CO_2_. Cells on the lower surface of the membrane were fixed with 4% paraformaldehyde for 20 min, stained with 0.1% crystal violet for 15 min, and visualized under a microscope.

### 4.8 HLECs Tube Formation Assays and Transwell Assays

Serum‐free ECM and Matrigel (BD Biosciences, CA, USA) were combined equally and then applied to 24‐well chambers, followed by incubation for at 37 ℃ overnight. Afterward, HLECs pretreated appropriately were seeded into the prepared chambers at a density of 1 × 10^5 cells per well. Chambers were supplemented with PBS control or various conditioned media (CM). Tube formation was observed by inverted fluorescence microscopy, and ImageJ software was used to measure the tube lengths.

### 4.9 HPLC‐Ms/Ms

Tryptic peptides were analyzed by nanoflow liquid chromatography–tandem mass spectrometry (nanoLC–MS/MS) using a Q Exactive HF‐X Orbitrap mass spectrometer coupled to an EASY‐nLC 1200 system (Thermo Fisher Scientific, USA) via a nano‐electrospray ionization source. Approximately 1 µg of peptides was loaded onto a 25 cm analytical column (100 µm inner diameter) packed with 1.5 µm ReproSil‐Pur C18‐AQ particles. Peptides were separated at a flow rate of 300 nL/min using a linear gradient of solvent B (80 % acetonitrile with 0.1 % formic acid) from 8 % to 40 % over 12 min, followed by a 10‐min wash at 95 % solvent B. Solvent A consisted of 0.1 % formic acid in water. Mass spectrometric data were acquired in data‐dependent acquisition mode, selecting the top 40 most intense precursor ions for fragmentation. Full MS scans were acquired in the Orbitrap at a resolution of 120,000 over an m/z range of 350‐1500. Precursor ions were isolated with a 1.6 m/z window and fragmented by higher‐energy collisional dissociation at a normalized collision energy of 27. MS/MS spectra were recorded at a resolution of 15,000, with dynamic exclusion set to 16 s.

### 4.10 ELISA

A Human ELISA Kit (Meimian Biotechnology Co., Ltd., China) was used following the manufacturer's specific protocol to measure the levels of CCL1, IL‐10, and VEGF‐C. In brief, tumor and bacterial culture supernatants from BCa or macrophage cells were harvested. The treated samples were then added to wells precoated with the specific antibody of CCL1, IL‐10, or VEGF‐C. After a 30 min incubation and coloration at 37°C, the absorbance of each well was recorded at 450 nm. The concentration present in each well was deduced based on a standard curve.

### 4.11 Cell Transfection

Lentiviral shRNA of shNC and shTLR2 were designed and synthesized by Miaoling Biology (Wuhan, China). The shRNA sequences for TLR2 were: TLR2‐sh1, 5'‐GCACACGAATACACAGTGTAA‐3', TLR2‐sh2, 5'‐GCGGAAGATAATGAACACCAA‐3'. Plasmid transfections into bladder cancer cell lines were performed using Lipofectamine 3000 reagent (Invitrogen), following the guidelines from the supplier. For 293T cell transfections, polyethyleneimine (PEI, Polysciences) was utilized according to the manufacturer's recommendations. Lentiviral particles were generated by co‐transfecting GFP shRNA plasmids with packaging plasmids (pMD2.G and psPAX2) into 293T cells. Target cells were infected using these viral particles in the presence of polybrene (10 µg/mL; Beyotime) for 8 h, followed by puromycin‐based selection to establish stable lines.

### 4.12 RNA Sequencing and Analysis

Cell supernatants were used for total RNA extraction with TRIzol reagent (Pricella Biotechnology Co., Ltd., Wuhan, China). RNA libraries were prepared using the KAPA RNA Library Prep Kit following the manufacturer's protocol and sequenced on the Illumina NovaSeq system. The mouse reference genome was sourced from the GENCODE database.

Differential gene expression analysis was conducted using the “edgeR” package in R, with significantly altered genes defined by a fold change (FC) greater than 1 and a p‐value below 0.05. KEGG pathway enrichment analysis of the differentially expressed genes (DEGs) was carried out, and the visualization of results was performed using the “enrichplot” R package.

### 4.13 Chromatin Immunoprecipitation (ChIP) Assay

Protein A/G magnetic beads were prepared following the instructions of MedChemExpress. The cells were harvested and washed before crosslinking with 1% formaldehyde for 10 min at room temperature, and 1.375 m glycine was added to neutralize them. The crosslinked cells were lysed on ice before being centrifuged at 1350×g for 5 min, and the supernatant was discarded. The samples were then sonicated using a noncontact ultrasonic disruptor. The DNA fragment size was checked by 2.5% agarose gel electrophoresis at 120 V for 15 min, with the desired size range being 200–600 bp.

Sheared DNA was divided into three aliquots for IP, IgG, and Input. The IP and IgG samples were incubated with antibody‐coupled beads, while the Input was stored at −20°C. DNA‐protein cross‐links were reversed by incubation at 65°C after being washed, followed by treatment with RNase A and Proteinase K. DNA was purified using phenol: chloroform: isoamyl alcohol extraction and ethanol precipitation. The enrichment of IL‐6 promoter regions was quantified by qPCR using specific primers and normalized to Input and IgG controls. The qPCR data were analyzed using the 2^‐ΔΔCt method to determine the relative enrichment of IL‐6 promoter sequences.

### 4.14 Western Blotting Analysis

Following treatment, bladder cancer cells were lysed using RIPA buffer (Cell Signaling Technology) supplemented with a protease and phosphatase inhibitor cocktail (New Cell & Molecular Biotech, Suzhou). Protein concentrations were normalized, and samples were resolved via SDS‐PAGE. Proteins were subsequently transferred to PVDF membranes. To minimize non‐specific binding, membranes were blocked in TBST containing 5% BSA. After blocking, membranes were incubated overnight at 4°C with the appropriate primary antibodies. The next day, membranes were washed three times with TBST (10 min each) and then exposed to secondary antibodies. A second round of TBST washes was performed before detection. Protein signals were developed using the GelView 6000 Pro imaging system (BLT, China). A list of antibodies used is provided in Table S2.

### 4.15 Immunofluorescence (IF) Staining

Immunofluorescence staining was performed following previously described protocols [60]. Briefly, BCa cells were seeded onto confocal dishes and fixed with 4% paraformaldehyde, followed by permeabilization with 0.5% Triton X‐100. The cells were then blocked and incubated overnight at 4°C with primary antibodies. After incubation, the dishes were washed three times with PBS and then incubated for 1 h at 25°C with secondary antibodies. Nuclear counterstaining was performed using DAPI (Beyotime, Shanghai, China) for 5 min at 25°C. Imaging was conducted using a confocal microscope (Zeiss, Munich, Germany).

### 4.16 Immunohistochemistry (IHC)

Immunohistochemistry was conducted as previously described [60]. Briefly, bladder cancer tissue sections, fixed in 10% formalin and paraffin‐embedded, were subjected to antigen retrieval with sodium citrate/EDTA after dewaxing and rehydration. The sections were then incubated overnight at 4°C with the primary antibody. The following day, the sections were treated with HRP‐labeled secondary antibody, developed with DAB, and counterstained with hematoxylin. Images were captured using a microscope and analyzed with ImageJ software. Molecular docking analysis.

### 4.17 Flow Cytometry Staining Procedure

Cells were counted and adjusted to 1×10⁶ cells/mL. After centrifugation (2,000 rpm, 5 min, 4 ℃), cells were sequentially incubated with Fc receptor blocking reagent (20 min, 4 ℃, dark) and Zombie viability dye (30 min, 4 ℃, dark), with a PBS wash between steps. Cells were then stained with fluorophore‐conjugated antibodies for surface markers in Flow Cytometry Staining Buffer II (30 min, 4 ℃, dark), washed, and subjected to intracellular staining using a Fixation/Permeabilization Kit according to the manufacturer’s instructions. Briefly, cells were fixed/permeabilized (20 min, 4 ℃, dark), washed with 1× Perm/Wash buffer, and incubated with intracellular antibodies (30 min, 4 ℃, dark). After final washing, cells were resuspended in Staining Buffer II and analyzed on a CytoFLEX flow cytometer (Beckman Coulter).

### 4.18 Protein and Compound Molecular Docking Screening

Multiple compound libraries were collected and preprocessed using OpenBabel. Compounds were converted from 2D to 3D structures, hydrogenated, assigned Gasteiger charges, energy‐minimized, and parameterized with the MMFF94 force field before being converted to PDBQT format. After removing duplicate compounds and those that failed structure generation or format conversion, a total of 50,932 compounds were retained for virtual screening. Compounds were primarily identified by CAS numbers, while those without CAS information were annotated using compound names, SMILES, or PubChem CIDs. The three‐dimensional structure of the target protein was predicted using AlphaFold3. High‐throughput virtual screening was first performed using AutoDock Vina. Compounds exhibiting unreasonable chemical structures or calculation errors were excluded. The top 5% of compounds ranked by binding affinity were selected for a second round of screening using AutoDock Vina, followed by a third round of refined screening on the top 5% of candidates from the second round to obtain the final binding affinity scores.

### 4.19 Bladder Cancer Organoid Culture

Fresh bladder cancer tumor samples were washed three times in PBS containing 50 µg/mL nystatin, 500 µg/mL streptomycin, and 500 U penicillin, and the samples were then cut into small particles and incubated with 1 mL of TrypLE for 45 min at 37°C. A 70‐mm cell strainer was used to remove undigested tissues, and the suspension was centrifuged to pellet the cells. The collected cells were resuspended in complete culture media mixed with a 1:2 volume of Matrigel matrix (BD Biosciences, Cat# 356234). Cells in droplets were plated in a 24‐well plate. Complete medium was added after the Matrigel polymerized, followed by culture at 37°C.

### 4.20 Statistical Analysis

GraphPad Prism 10.0 was used for statistical analysis, and the data are presented as means ± SEM. The Mann–Whitney U‐test was used to analyze nonparametric data, with the significance threshold set at *p*<0.05. While one‐way ANOVA, along with Tukey's test, was used for multiple group comparisons. As shown in the figure legend, significance levels are marked as ^*^ *p*<0.05, ^**^ *p*<0.01, and ^***^ *p*<0.001; ns indicates no significant difference.

## Funding

This work was supported by The National Natural Science Foundation of China (82503289); President Foundation of Nanfang Hospital, Southern Medical University (2024A028), China Postdoctoral Science Foundation (2025M782052) to Bisheng Cheng. National Natural Science Foundation of China (82570912, 81870522, and 82173304), Guangzhou Key Research and Development Program (2023B03J1245) to Peng Wu.

## Conflicts of Interest

The authors declare no conflicts of interest.

## Ethics Statement

All research protocols were approved by the Ethics Committee of Nanfang Hospital, Southern Medical University (IACUC‐LAC‐20240510‐004).

## Supporting information




**Supporting File**: advs74256‐sup‐0001‐SuppMat.docx.

## Data Availability

Research data are not shared.

## References

[1] A. G. Van Der Heijden , H. M. Bruins , and A. Carrion , “European Association of Urology Guidelines on Muscle‐Invasive and Metastatic Bladder Cancer: Summary of the 2025 Guidelines,” European Urology 87, no. 5 (2025): 582–600, 10.1016/j.eururo.2025.02.019. 40118736

[2] I. Jubber , S. Ong , L. Bukavina , et al., “Epidemiology of Bladder Cancer in 2023: a Systematic Review of Risk Factors,” European Urology 84, no. 2: 176–190, 10.1016/j.eururo.2023.03.029. 37198015

[3] Q. Zhang , S. Liu , H. Wang , et al., “ETV4 Mediated Tumor‐Associated Neutrophil Infiltration Facilitates Lymphangiogenesis and Lymphatic Metastasis of Bladder Cancer,” Advanced Science 10, no. 11 (2023): 2205613, 10.1002/advs.202205613. 36670069 PMC10104629

[4] K. Xiao , S. Peng , J. Lu , et al., “UBE2S interacting with TRIM21 Mediates the K11‐linked Ubiquitination of LPP to Promote the Lymphatic Metastasis of Bladder Cancer,” Cell Death & Disease 14, no. 7 (2023): 408, 10.1038/s41419-023-05938-2. 37422473 PMC10329682

[5] L. Cheng , C. Yang , J. Lu , et al., “Oncogenic SLC2A11–MIF Fusion Protein Interacts with Polypyrimidine Tract Binding Protein 1 to Facilitate Bladder Cancer Proliferation and Metastasis by Regulating mRNA Stability,” MedComm 5, no. 9 (2024): 685, 10.1002/mco2.685. PMC11324686 39156764

[6] A. J. Wolfe , E. Toh , N. Shibata , et al., “Evidence of Uncultivated Bacteria in the Adult Female Bladder,” Journal of Clinical Microbiology 50, no. 4 (2012): 1376–1383, 10.1128/JCM.05852-11. 22278835 PMC3318548

[7] S. A. Whiteside , H. Razvi , S. Dave , G. Reid , and J. P. Burton , “The Microbiome of the Urinary Tract—A Role beyond Infection,” Nature Reviews Urology 12, no. 2 (2015): 81–90, 10.1038/nrurol.2014.361. 25600098

[8] E. Shrestha , J. R. White , S.‐H. Yu , et al., “Profiling the Urinary Microbiome in Men with Positive versus Negative Biopsies for Prostate Cancer,” Journal of Urology 199, no. 1 (2018): 161–171, 10.1016/j.juro.2017.08.001. 28797714 PMC5937117

[9] P. Wu , Y. Chen , J. Zhao , et al., “Urinary Microbiome and Psychological Factors in Women with Overactive Bladder,” Frontiers in Cellular and Infection Microbiology 7, (2017): 488, 10.3389/fcimb.2017.00488. 29230385 PMC5712163

[10] G. Serna , F. Ruiz‐Pace , J. Hernando , et al., “Fusobacterium nucleatum Persistence and Risk of Recurrence after Preoperative Treatment in Locally Advanced Rectal Cancer,” Annals of Oncology 31, no. 10 (2020): 1366–1375, 10.1016/j.annonc.2020.06.003. 32569727 PMC7542577

[11] L. Parhi , T. Alon‐Maimon , A. Sol , et al., “Breast Cancer Colonization by Fusobacterium nucleatum Accelerates Tumor Growth and Metastatic Progression,” Nature Communications 11, no. 1 (2020): 3259, 10.1038/s41467-020-16967-2. PMC7320135 32591509

[12] A. Audirac‐Chalifour , K. Torres‐Poveda , M. Bahena‐Román , et al., “Cervical Microbiome and Cytokine Profile at Various Stages of Cervical Cancer: a Pilot Study,” PLoS ONE 11, no. 4 (2016): 0153274, 10.1371/journal.pone.0153274. PMC4846060 27115350

[13] H. Zhao , M. Chu , Z. Huang , et al., “Variations in Oral Microbiota Associated with Oral Cancer,” Scientific Reports 7, no. 1 (2017): 11773, 10.1038/s41598-017-11779-9. 28924229 PMC5603520

[14] V. Bučević Popović , M. Šitum , C. E. T. Chow , et al., “The Urinary Microbiome Associated with Bladder Cancer,” Scientific Reports 8, no. 1 (2018): 12157, 10.1038/s41598-018-29054-w. 30108246 PMC6092344

[15] C. Schwechheimer and M. J. Kuehn , “Outer‐membrane Vesicles from Gram‐negative Bacteria: Biogenesis and Functions,” Nature Reviews Microbiology 13, no. 10 (2015): 605–619, 10.1038/nrmicro3525. 26373371 PMC5308417

[16] X. Zheng , T. Gong , W. Luo , et al., “Fusobacterium nucleatum Extracellular Vesicles Are Enriched in Colorectal Cancer and Facilitate Bacterial Adhesion,” Science Advances 10, no. 38 (2024): ado0016, 10.1126/sciadv.ado0016. PMC11414721 39303027

[17] J. Chew , P. S. Zilm , J. M. Fuss , and N. J. Gully , “A Proteomic Investigation of Fusobacterium nucleatum Alkaline‐induced Biofilms,” BMC Microbiology 12, (2012): 189, 10.1186/1471-2180-12-189. 22943491 PMC3478200

[18] E.‐H. Hwanga , T.‐H. Kim , J.‐Y. Park , et al., “TLR2 contributes to Trigger Immune Response of Pleural Mesothelial Cells against Mycobacterium Bovis BCG and M. tuberculosis Infection,” Cytokine 95, (2017): 80–87, 10.1016/j.cyto.2017.02.021. 28249177

[19] C. Martin‐Gallausiaux , A. Malabirade , J. Habier , and P. Wilmes , “Fusobacterium nucleatum Extracellular Vesicles Modulate Gut Epithelial Cell Innate Immunity via FomA and TLR2,” Frontiers in Immunology 11, (2020): 583644, 10.3389/fimmu.2020.583644. 33408714 PMC7779620

[20] I. H. Han , H. O. Song , and J. S. Ryu , “IL‐6 Produced by Prostate Epithelial Cells Stimulated with Trichomonas vaginalis Promotes Proliferation of Prostate Cancer Cells by Inducing M2 Polarization of THP‐1‐derived Macrophages,” PLoS Neglected Tropical Diseases 14, no. 3 (2020): 0008126, 10.1371/journal.pntd.0008126. PMC7138318 32196489

[21] L. Browning , M. Patel , E. Bring Horvath , K. Tawara , and C. L. Jorcyk , “IL‐6 and Ovarian Cancer: Inflammatory Cytokines in Promotion of Metastasis,” Cancer Management and Research 10, (2018): 6685–6693, 10.2147/CMAR.S179189. 30584363 PMC6287645

[22] T. Ara and Y. A. Declerck , “Interleukin‐6 in Bone Metastasis and Cancer Progression,” European Journal of Cancer 46, no. 7 (1990): 1223–1231, 10.1016/j.ejca.2010.02.026. PMC2917917 20335016

[23] W.‐Q. Du , Z.‐M. Zhu , X. Jiang , M.‐J. Kang , and D.‐S. Pei , “COPS6 promotes Tumor Progression and Reduces CD8+ T Cell Infiltration by Repressing IL‐6 Production to Facilitate Tumor Immune Evasion in Breast Cancer,” Acta Pharmacologica Sinica 44, no. 9 (2023): 1890–1905, 10.1038/s41401-023-01085-8. 37095198 PMC10462724

[24] Y.‐S. Weng , H.‐Y. Tseng , Y.‐A. Chen , et al., “MCT‐1/miR‐34a/IL‐6/IL‐6R Signaling Axis Promotes EMT Progression, Cancer Stemness and M2 Macrophage Polarization in Triple‐negative Breast Cancer,” Molecular Cancer 18, no. 1 (2019): 42, 10.1186/s12943-019-0988-0. 30885232 PMC6421700

[25] S. T. Orange , J. Leslie , M. Ross , D. A. Mann , and H. Wackerhage , “The Exercise IL‐6 Enigma in Cancer,” Trends in Endocrinology & Metabolism 34, no. 11 (2023): 749–763, 10.1016/j.tem.2023.08.001. 37633799

[26] M. Locati , G. Curtale , and A. Mantovani , “Diversity, Mechanisms, and Significance of Macrophage Plasticity,” Annual Review of Pathology: Mechanisms of Disease 15, no. 1 (2020): 123–147, 10.1146/annurev-pathmechdis-012418-012718. PMC7176483 31530089

[27] S. F. Schoppmann , P. Birner , J. Stöckl , et al., “Tumor‐Associated Macrophages Express Lymphatic Endothelial Growth Factors and Are Related to Peritumoral Lymphangiogenesis,” The American Journal of Pathology 161, no. 3 (2002): 947–956, 10.1016/S0002-9440(10)64255-1. 12213723 PMC1867252

[28] C. Xu , L. Fan , Y. Lin , et al., “Fusobacterium nucleatum Promotes Colorectal Cancer Metastasis through miR‐1322/CCL20 Axis and M2 Polarization,” Gut Microbes 13, no. 1 (2021): 1980347, 10.1080/19490976.2021.1980347. 34632963 PMC8510564

[29] F. Nie , J. Zhang , H. Tian , et al., “The Role of CXCL2‐mediated Crosstalk between Tumor Cells and Macrophages in Fusobacterium nucleatum‐promoted Oral Squamous Cell Carcinoma Progression,” Cell Death & Disease 15, no. 4 (2024): 277, 10.1038/s41419-024-06640-7. 38637499 PMC11026399

[30] M. A. Casasanta , C. C. Yoo , B. Udayasuryan , et al., “Fusobacterium nucleatum Host‐cell Binding and Invasion Induces IL‐8 and CXCL1 Secretion That Drives Colorectal Cancer Cell Migration,” Science Signaling 13, no. 641 (2020): aba9157, 10.1126/scisignal.aba9157. PMC7454160 32694172

[31] L.‐X. Wang , S.‐X. Zhang , H.‐J. Wu , X.‐L. Rong , and J. Guo , “M2b macrophage Polarization and Its Roles in Diseases,” Journal of Leukocyte Biology 106, no. 2 (2019): 345–358, 10.1002/JLB.3RU1018-378RR. 30576000 PMC7379745

[32] A. Mantovani , F. Marchesi , A. Malesci , L. Laghi , and P. Allavena , “Tumour‐Associated Macrophages as Treatment Targets in Oncology,” Nature Reviews Clinical Oncology 14, no. 7 (2017): 399–416, 10.1038/nrclinonc.2016.217. PMC5480600 28117416

[33] J. Zeng , G. Zhang , C. Chen , et al., “Alterations in Urobiome in Patients with Bladder Cancer and Implications for Clinical Outcome: a Single‐Institution Study,” Frontiers in Cellular and Infection Microbiology 10, (2020): 555508, 10.3389/fcimb.2020.555508. 33384966 PMC7769872

[34] M. C. Markowski , S. A. Boorjian , J. P. Burton , et al., “The Microbiome and Genitourinary Cancer: a Collaborative Review,” European Urology 75, no. 4 (2019): 637–646, 10.1016/j.eururo.2018.12.043. 30655087 PMC9774685

[35] F. Liedberg and W. Månsson , “Lymph Node Metastasis in Bladder Cancer,” European Urology 49, no. 1 (2006): 13–21, 10.1016/j.eururo.2005.08.007. 16203077

[36] S. Chen , L. Zhang , M. Li , et al., “Fusobacterium nucleatum Reduces METTL3‐mediated m6A Modification and Contributes to Colorectal Cancer Metastasis,” Nature Communications 13, no. 1 (2022): 1248, 10.1038/s41467-022-28913-5. PMC8913623 35273176

[37] A. Fu , B. Yao , T. Dong , et al., “Tumor‐Resident Intracellular Microbiota Promotes Metastatic Colonization in Breast Cancer,” Cell 185, no. 8 (2022): 1356–1372.e26, 10.1016/j.cell.2022.02.027. 35395179

[38] L. T. Geller , M. Barzily‐Rokni , T. Danino , et al., “Potential Role of Intratumor Bacteria in Mediating Tumor Resistance to the Chemotherapeutic Drug Gemcitabine,” Science 357, no. 6356 (2017): 1156–1160, 10.1126/science.aah5043. 28912244 PMC5727343

[39] G. Chen , C. Gao , S. Jiang , et al., “Fusobacterium nucleatum Outer Membrane Vesicles Activate Autophagy to Promote Oral Cancer Metastasis,” Journal of Advanced Research 56, (2024): 167–179, 10.1016/j.jare.2023.04.002. 37059221 PMC10834801

[40] S. Wang , A. Song , J. Xie , et al., “Fn‐OMV Potentiates ZBP1‐mediated PANoptosis Triggered by Oncolytic HSV^−1^ to Fuel Antitumor Immunity,” Nature Communications 15, no. 1 (2024): 3669, 10.1038/s41467-024-48032-7. PMC11063137 38693119

[41] H. Wang , H. Zhan , B. Pan , et al., “Engineering CRISPR System‐Based Bacterial Outer Membrane Vesicle Potentiates T Cell Immunity for Enhanced Cancer Immunotherapy,” Advanced Materials 37, no. 39 (2025): 2501565, 10.1002/adma.202501565. 40495695 PMC12506603

[42] Q.‐Q. Chai , D. Li , M. Zhang , et al., “Engineering Nanoplatforms of Bacterial Outer Membrane Vesicles to Overcome Cancer Therapy Resistance,” Drug Resistance Updates 83, (2025): 101277, 10.1016/j.drup.2025.101277. 40712413

[43] W. Li , Z. Zhang , R. Wu , et al., “Fusobacterium nucleatum ‐Derived Outer Membrane Vesicles Promote Immunotherapy Resistance via Changes in Tryptophan Metabolism in Tumour‐Associated Macrophages,” Journal of Extracellular Vesicles 14, no. 4 (2025): 70070, 10.1002/jev2.70070. PMC12003102 40241230

[44] X. Li , X. Li , and J. Shi , “Engineered Outer Membrane Vesicles Enhance Solid Tumour CAR‐T Cell Therapy,” Nature Biomedical Engineering (2026), 10.1038/s41551-025-01575-6. PMC13099647 41501251

[45] M. Toyofuku , N. Nomura , and L. Eberl , “Types and Origins of Bacterial Membrane Vesicles,” Nature Reviews Microbiology 17, no. 1 (2019): 13–24, 10.1038/s41579-018-0112-2. 30397270

[46] P. K. Bista , D. Pillai , and S. K. Narayanan , “Outer‐Membrane Vesicles of Fusobacterium Necrophorum: a Proteomic, Lipidomic, and Functional Characterization,” Microorganisms 11, no. 8 (2023): 2082, 10.3390/microorganisms11082082. 37630642 PMC10458137

[47] Z. Zhang , S. Liu , S. Zhang , et al., “Porphyromonas gingivalis Outer Membrane Vesicles Inhibit the Invasion of Fusobacterium nucleatum into Oral Epithelial Cells by Downregulating FadA and FomA,” Journal of Periodontology 93, no. 4 (2022): 515–525, 10.1002/JPER.21-0144. 34458990 PMC9415117

[48] D. N. Toussi , X. Liu , and P. Massari , “The FomA Porin from Fusobacterium nucleatum Is a Toll‐Like Receptor 2 Agonist with Immune Adjuvant Activity,” Clinical and Vaccine Immunology 19, no. 7 (2012): 1093–1101, 10.1128/CVI.00236-12. 22623652 PMC3393365

[49] D. E. Johnson , R. A. O'Keefe , and J. R. Grandis , “Targeting the IL‐6/JAK/STAT3 Signalling Axis in Cancer,” Nature Reviews Clinical Oncology 15, no. 4 (2018): 234–248, 10.1038/nrclinonc.2018.8. PMC5858971 29405201

[50] M. Rašková , L. Lacina , and Z. Kejík , “The Role of IL‐6 in Cancer Cell Invasiveness and Metastasis‐Overview and Therapeutic Opportunities,” Cells 11, no. 22 (2022): 3698, 10.3390/cells11223698. 36429126 PMC9688109

[51] S. Janssens and R. Beyaert , “Role of Toll‐Like Receptors in Pathogen Recognition,” Clinical Microbiology Reviews 16, no. 4 (2003): 637–646, 10.1128/CMR.16.4.637-646.2003. 14557290 PMC207104

[52] C. L. Pocanschi , H.‐J. Apell , P. Puntervoll , et al., “The Major Outer Membrane Protein of Fusobacterium nucleatum (FomA) Folds and Inserts into Lipid Bilayers via Parallel Folding Pathways,” Journal of Molecular Biology 355, no. 3 (2006): 548–561, 10.1016/j.jmb.2005.10.060. 16310217

[53] R. Yang , Y. Liao , L. Wang , et al., “Exosomes Derived from M2b Macrophages Attenuate DSS‐Induced Colitis,” Frontiers in Immunology 10, (2019): 2346, 10.3389/fimmu.2019.02346. 31749791 PMC6843072

[54] D. Philipp , L. Suhr , T. Wahlers , Y.‐H. Choi , and A. Paunel‐Görgülü , “Preconditioning of Bone Marrow‐derived Mesenchymal Stem Cells Highly Strengthens Their Potential to Promote IL‐6‐dependent M2b Polarization,” Stem Cell Research & Therapy 9, no. 1 (2018): 286, 10.1186/s13287-018-1039-2. 30359316 PMC6202843

[55] R. Noy and J. W. Pollard , “Tumor‐Associated Macrophages: from Mechanisms to Therapy,” Immunity 41, no. 1 (2014): 49–61, 10.1016/j.immuni.2014.06.010. 25035953 PMC4137410

[56] P. Pathria , T. L. Louis , and J. A. Varner , “Targeting Tumor‐Associated Macrophages in Cancer,” Trends in Immunology 40, no. 4 (2019): 310–327, 10.1016/j.it.2019.02.003. 30890304

[57] M. Huang , W. Dong , R. Xie , et al., “HSF1 facilitates the Multistep Process of Lymphatic Metastasis in Bladder Cancer via a Novel PRMT5‐WDR5‐Dependent Transcriptional Program,” Cancer Communications 42, no. 5 (2022): 447–470, 10.1002/cac2.12284. 35434944 PMC9118058

[58] H. Gong , “Pinocembrin Suppresses Proliferation and Enhances Apoptosis in Lung Cancer Cells in Vitro by Restraining Autophagy,” Bioengineered 12, no. 1 (2021): 6035–6044, 10.1080/21655979.2021.1972779. 34486470 PMC8806703

[59] J. Gao , S. Lin , Y. Gao , et al., “Pinocembrin Inhibits the Proliferation and Migration and Promotes the Apoptosis of Ovarian Cancer Cells through Down‐regulating the mRNA Levels of N‐Cadherin and GABAB Receptor,” Biomedicine & Pharmacotherapy 120, (2019): 109505, 10.1016/j.biopha.2019.109505. 31634778

[60] W. Li , W. Shangguan , and W. Huang , “Gut Parabacteroides Distasonis‐derived Indole‐3‐Acetic Acid Promotes Phospholipid Remodeling and Enhances Ferroptosis Sensitivity via the AhR‐FASN Axis in Bladder Cancer,” Advanced Science 12, (2025): 04688, 10.1002/advs.202504688. PMC12442663 40557796

