# Supplementary material for: FomA‐Containing Outer Membrane Vesicles of Fusobacterium Nucleatum Facilitate Bladder Cancer Lymphatic Metastasis via IL‐6‐Dependent M2b Macrophage Polarization
Source: Adv Sci (Weinh). 2026 Feb 13;13(23):e23256. doi: 10.1002/advs.202523256 (PMC13104081; doi:10.1002/advs.202523256)
Supplement: Supplementary file 1 — Supporting File: advs74256‐sup‐0001‐SuppMat.docx. [file ADVS-13-e23256-s001.docx]

### Supplementary Materials

**Supplementary Table****s**

**Supplementary Table S1** The primer sequences applied in this paper are as follows:

| Gene | Forward primer (5ʹ–3ʹ) | Reverse primer (5ʹ–3ʹ) |
| --- | --- | --- |
| GAPDH (hum) | GGAGCGAGATCCCTCCAAAAT | GGCTGTTGTCATACTTCTCATGG |
| GAPDH (mou) | CCTGACCTGCCGTCTAGAAA | CTCCGACGCCTGCTTCAC |
| TLR2 (hum) | CTTCACTCAGGAGCAGCAAGCA | ACACCAGTGCTGTCCTGTGACA |
| IL-6 (hum) | AGACAGCCACTCACCTCTTCAG | TTCTGCCAGTGCCTCTTTGCTG |
| NF-κB1 (hum) | GCAGCACTACTTCTTGACCACC | TCTGCTCCTGAGCATTGACGTC |
| CSF2 (hum) | GGAGCATGTGAATGCCATCCAG | CTGGAGGTCAAACATTTCTGAGAT |
| CXCL1 (hum) | AGCTTGCCTCAATCCTGCATCC | TCCTTCAGGAACAGCCACCAGT |
| CXCL2 (hum) | GGCAGAAAGCTTGTCTCAACCC | CTCCTTCAGGAACAGCCACCAA |
| CCL20 (hum) | AAGTTGTCTGTGTGCGCAAATCC | CCATTCCAGAAAAGCCACAGTTTT |
| CCL1 (mou) | GCTTACGGTCTCCAATAGCTGC | GCTTTCTCTACCTTTGTTCAGCC |
| IL-10 (hum) | TCTCCGAGATGCCTTCAGCAGA | TCAGACAAGGCTTGGCAACCCA |
| IL-6 (mou) | TACCACTTCACAAGTCGGAGGC | CTGCAAGTGCATCATCGTTGTTC |
| CCL1 (hum) | ACCAGCTCCATCTGCTCCAATG | TGTGCCTCTGAACCCATCCAAC |
| IL-10 (mou) | CGGGAAGACAATAACTGCACCC | CGGTTAGCAGTATGTTGTCCAGC |
| VEGFC (hum) | GAGGAGCAGTTACGGTCTGTG | TCCTTTCCTTAGCTGACACTTGT |
| Chip IL-6 | CGTCACATTGCACAATCTTA | TCAGACATCTCCAGTCCTATA |

**Supplementary Table S2** Antibodies used in this study

| Protein | SOURCE | Cat number |
| --- | --- | --- |
| Monoclonal [TL2.1] to TLR2 | Abcam | ab9100 |
| GAPDH Monoclonal antibody | Proteintech | Cat No. 60004-1-Ig |
| NF kappaB p105/p50 Antibody | Affinity Bioscience | AF6217 |
| Phospho-NF kappaB p105/p50 (Ser337) Antibody | Affinity Bioscience | AF3219 |
| Phospho-IKK alpha/ beta (Ser180/Ser181) Antibody | Affinity Bioscience | AF3013 |
| IL-6 Polyclonal antibody | Proteintech | Cat No. 21865-1-AP |
| Rabbit Polyclonal anti-FomA | This paper | N/A |
| FITC anti-mouse/human CD11b | BioLegend | Cat# 101205 |
| PE anti-mouse F4/80 | BioLegend | Cat# 123109 |
| hCCL1/I-309 Aff Pur (25 ug) | R&D | AF272-SP |
| mCCL1/TCA-3 Aff Pur (25 ug) | R&D | AF845-SP |
| Donkey anti-goat IgG H&L pre-adsorbed secondary antibody | Abcam | ab175665 |
| APC/Cyanine7 anti-mouse IL-10 | BioLegend | Cat# 505035 |
| PE anti-human IL-10 Antibody | BioLegend | Cat# 506804 |
| IL-10 Antibody | Abcam | ab9969 |

**Supplementary Tables S3** Top 10 compounds of Autodock Vina analysis

| **NO.** | **Compound** | **Max Affinity (kcal/mol)** | **CAS NO.** |
| --- | --- | --- | --- |
| 1 | Nosiheptide | -12.27 | 56377-79-8 |
| 2 | Pinocembrin | -11.48 | 205370-59-8 |
| 3 | Hyperwightin B | -11.27 | 2374723-86-9 |
| 4 | Asperazine | -11.21 | 198953-76-3 |
| 5 | alpha-Solamarine | -11.2 | 20318-30-3 |
| 6 | Punicalagin | -11.18 | 65995-63-3 |
| 7 | Zingiberen newsaponin | -10.99 | 91653-50-8 |
| 8 | Berninamycin C | -10.98 | 161263-49-6 |
| 9 | Deltonin | -10.93 | 55659-75-1 |
| 10 | Chonglou Saponin VII | -10.93 | 68124-04-9 |

**Supplementary Figures**


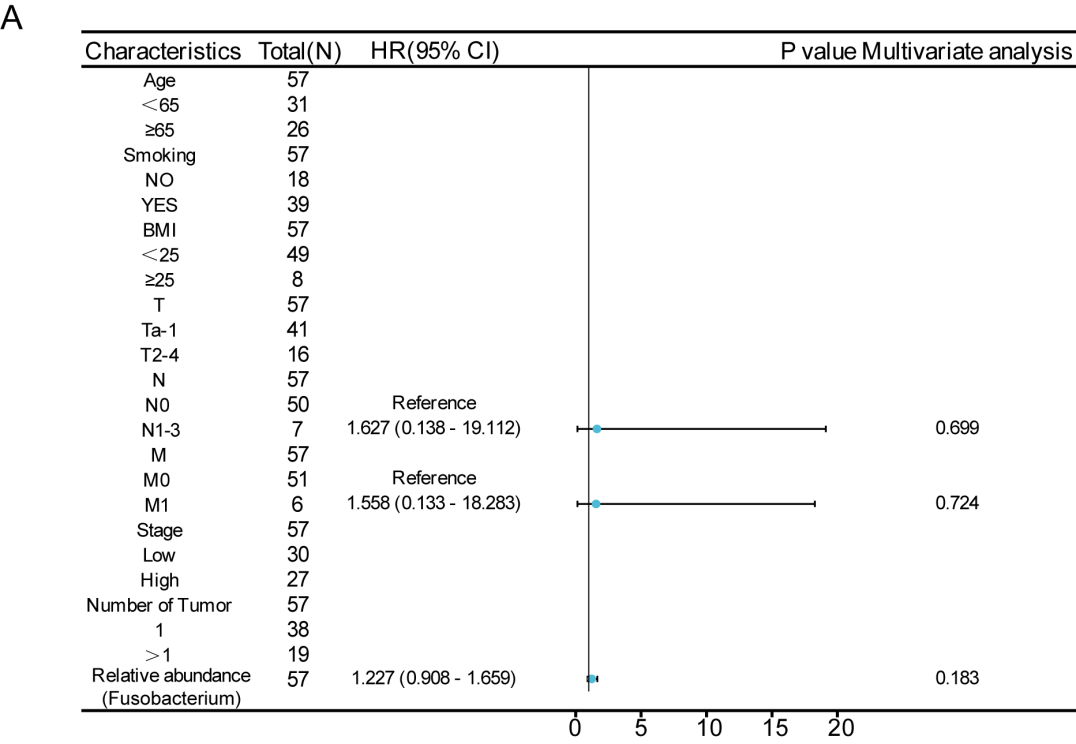


**Figure S1**

Multivariate COX regression analysis of different characteristics of BCa patients


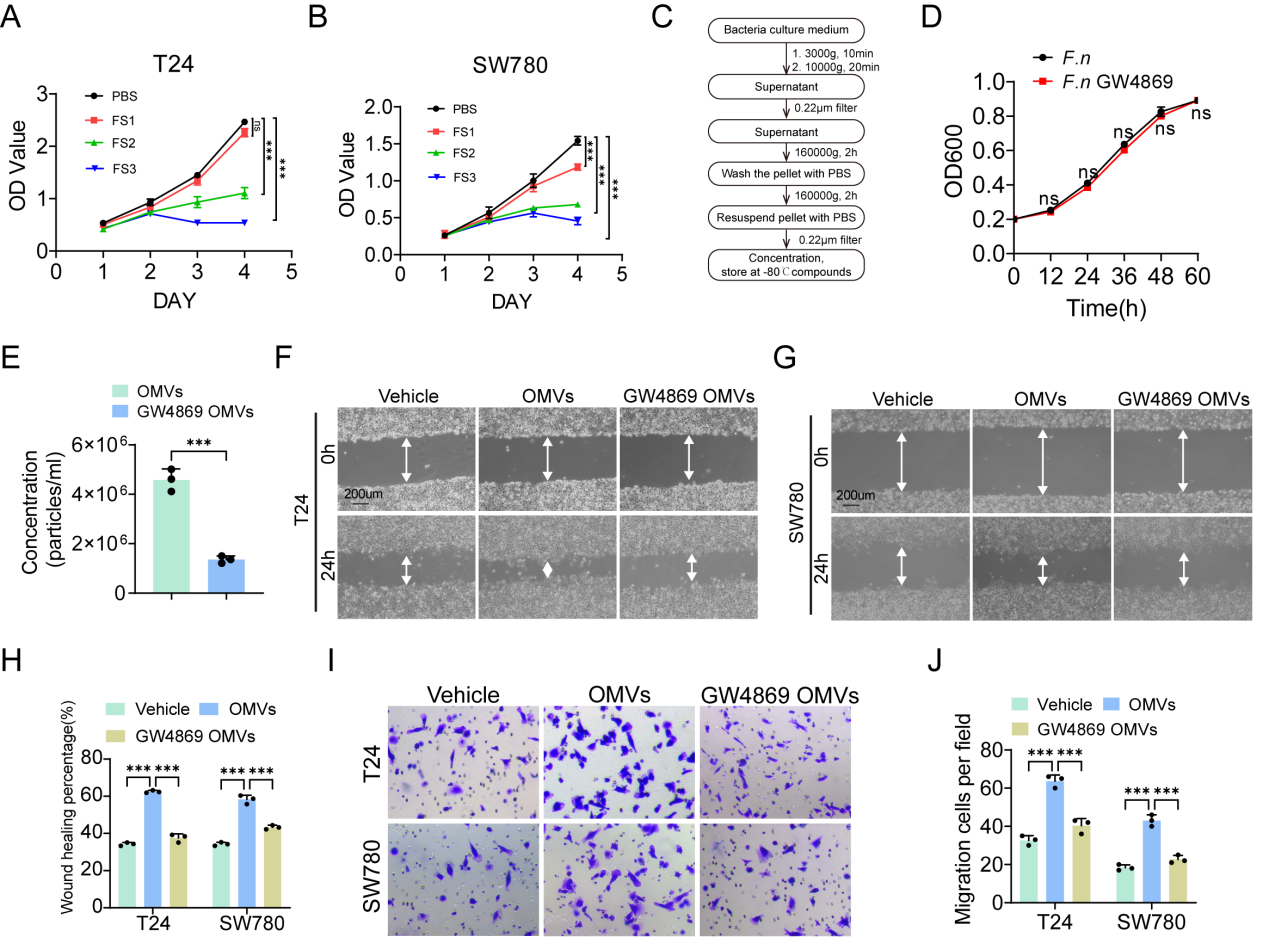


**Figure S2**

1. nucleatum supernatants and GW4869 treated F.n OMVs inhibit the migration of BCa cells. (A-B) Cell viability was evaluated by CCK8 in T24 and SW780 cells. (C) Nanoparticle concentration of F. n OMVs in control and GW4869-treated groups. (D) Growth curves of F.n in control and GW4869-treated conditions; OD600 indicates optical density at 600 nm. (E-F) Representative wound-healing assay images of T24 and SW780 cells under different treatments. (G) Quantification of wound closure. (H-J) Representative images and quantification of migrated cells in T24 and SW780 cells under different treatments. FS: *F.nucleatum* supernatants. FS1: FS (1%). FS2: FS (3%), FS3: FS (5%).


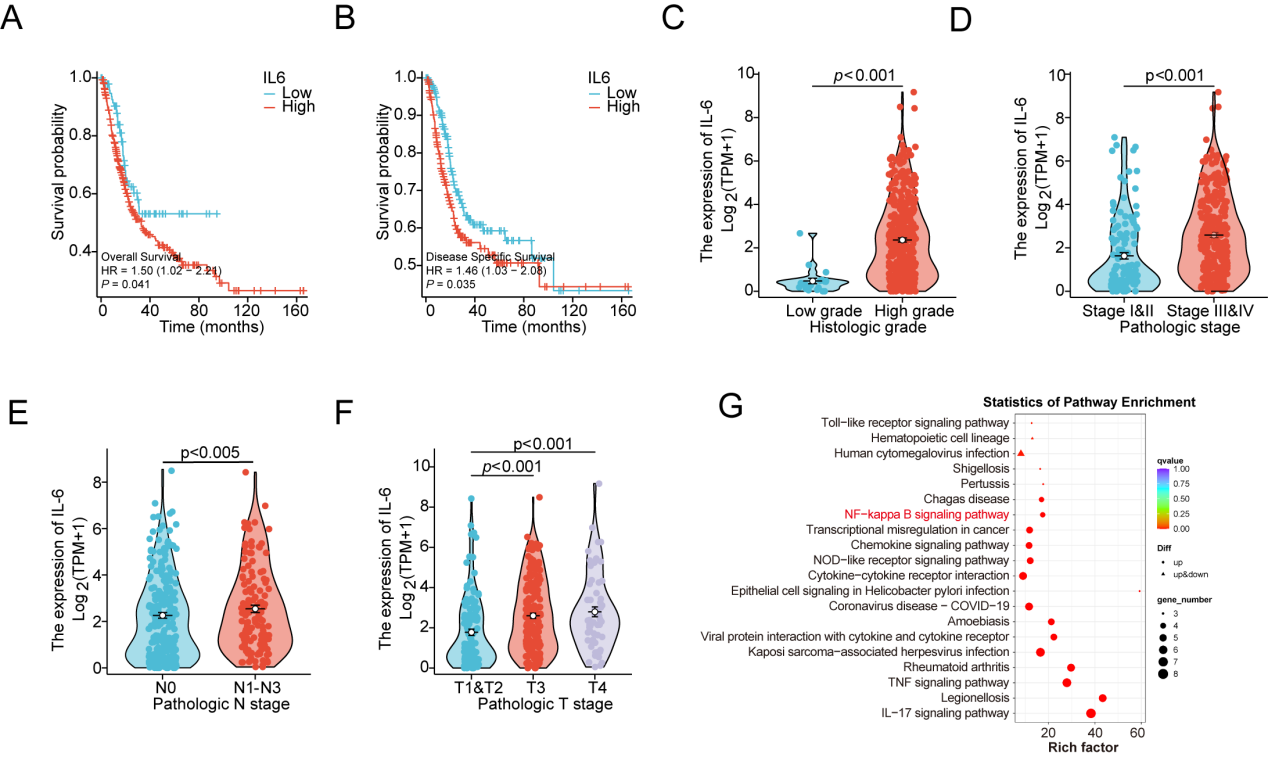


**Figure S3**

IL-6 of is associated with the stage and grade of BCa patients in TCGA database. (A-F) Association between IL-6 expression and bladder cancer prognosis, stage, and grade based on TCGA data. (G) NF-kappa B signaling pathway is enriched in KEGG pathway from RNA sequence.


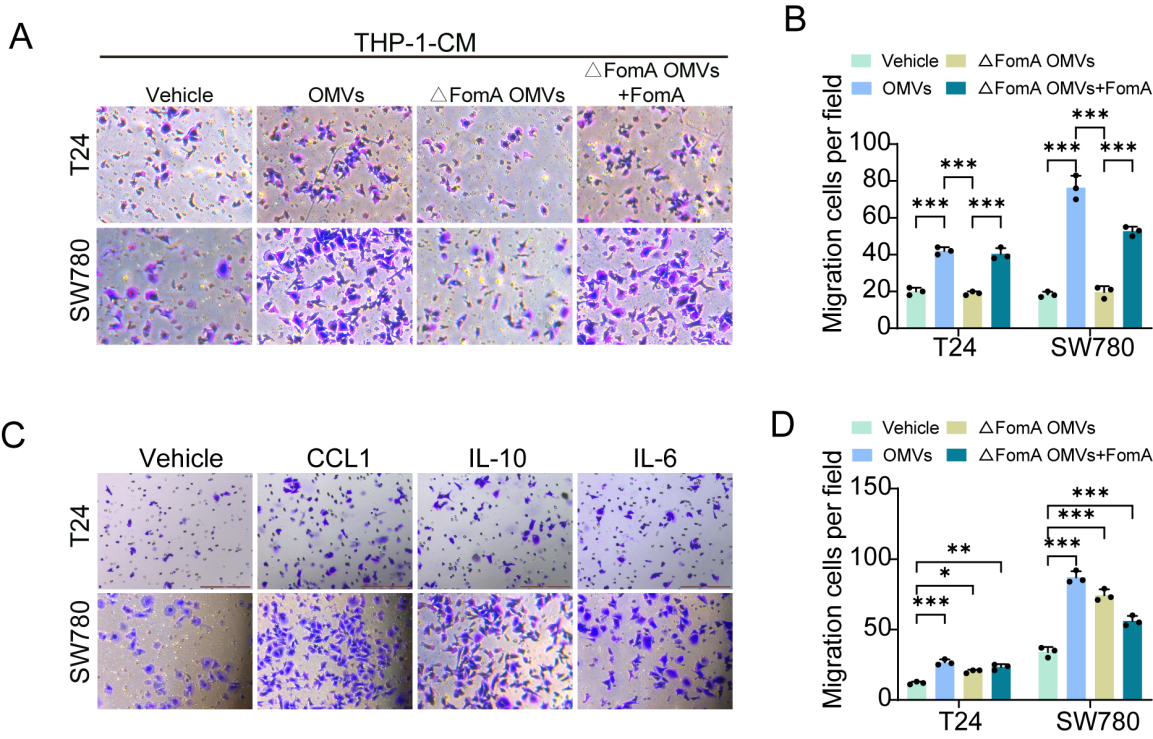


**Figure S4**

Conditioned medium from Macrophage treated by OMVs or FomA promote the migration of BCa cell. (A-B) Representative image of transwell migration from T24 and SW780 cells treated by different THP-1 CM. (C-D) Representative image of transwell migration from T24 and SW780 cells treated by ccl1, IL-6, and IL-10. CM: Conditional Medium


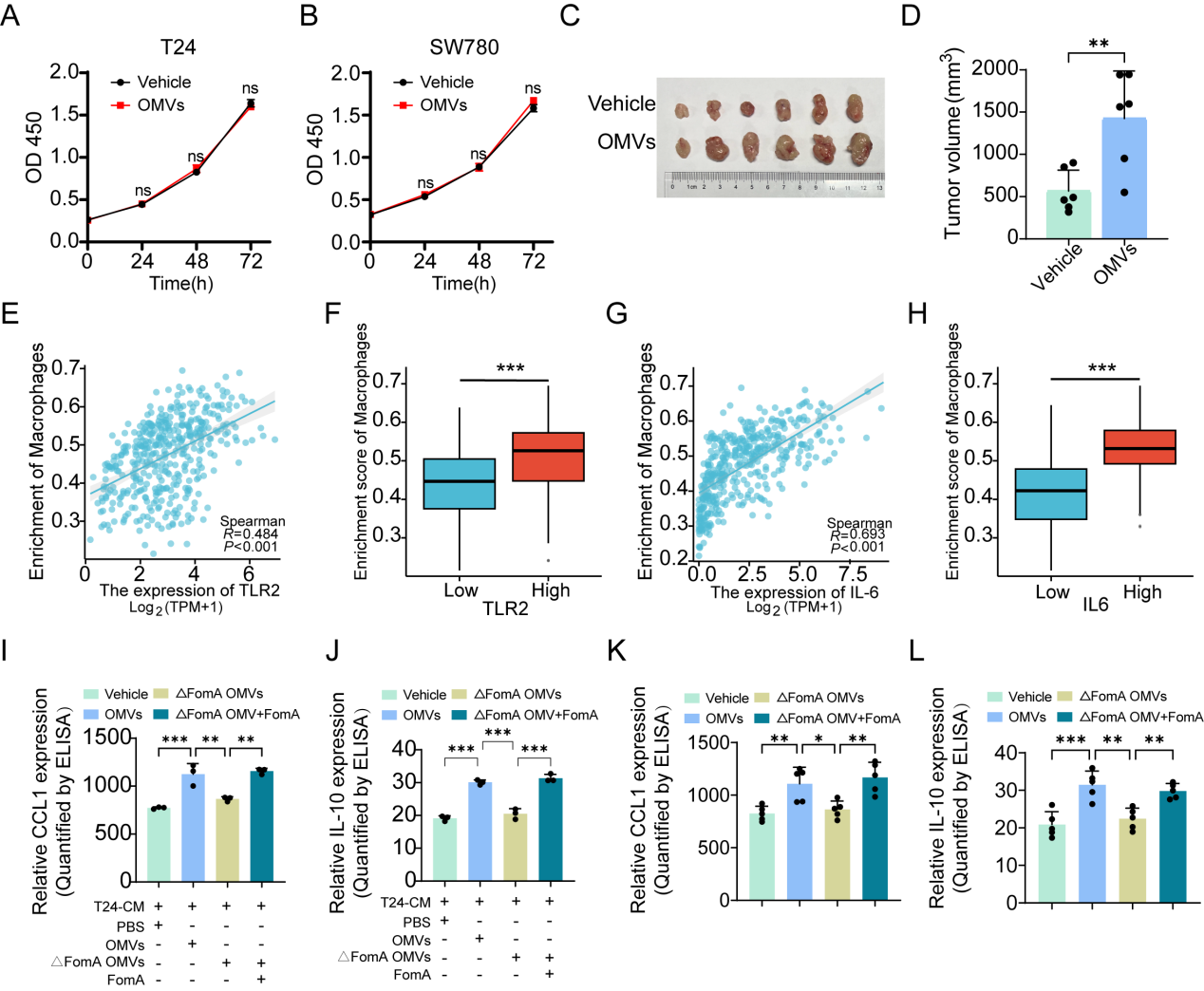


**Figure S5**

FomA-enriched OMVs from F.nucleatum facilitate M2b macrophage polarization. Conditioned medium from Macrophage treated by OMVs or FomA promote the migration of BCa cell. (A-B) Cell viability was evaluated by CCK8 in T24 and SW780 cells. (C-D) Images and volume of bladder tumors from the subcutaneous tumor model established in BALB/c nude mice. (E-F) Correlation analysis between TLR2 expression and macrophage infiltration in the TCGA dataset. (G-H) Correlation analysis between IL-6 expression and macrophage infiltration. (I-J) ELISA analysis of CCL1 and IL-10 levels of PBMC. (K-L) ELISA analysis of CCL1 and IL-10 levels of TAMs of Mice tumor tissues.


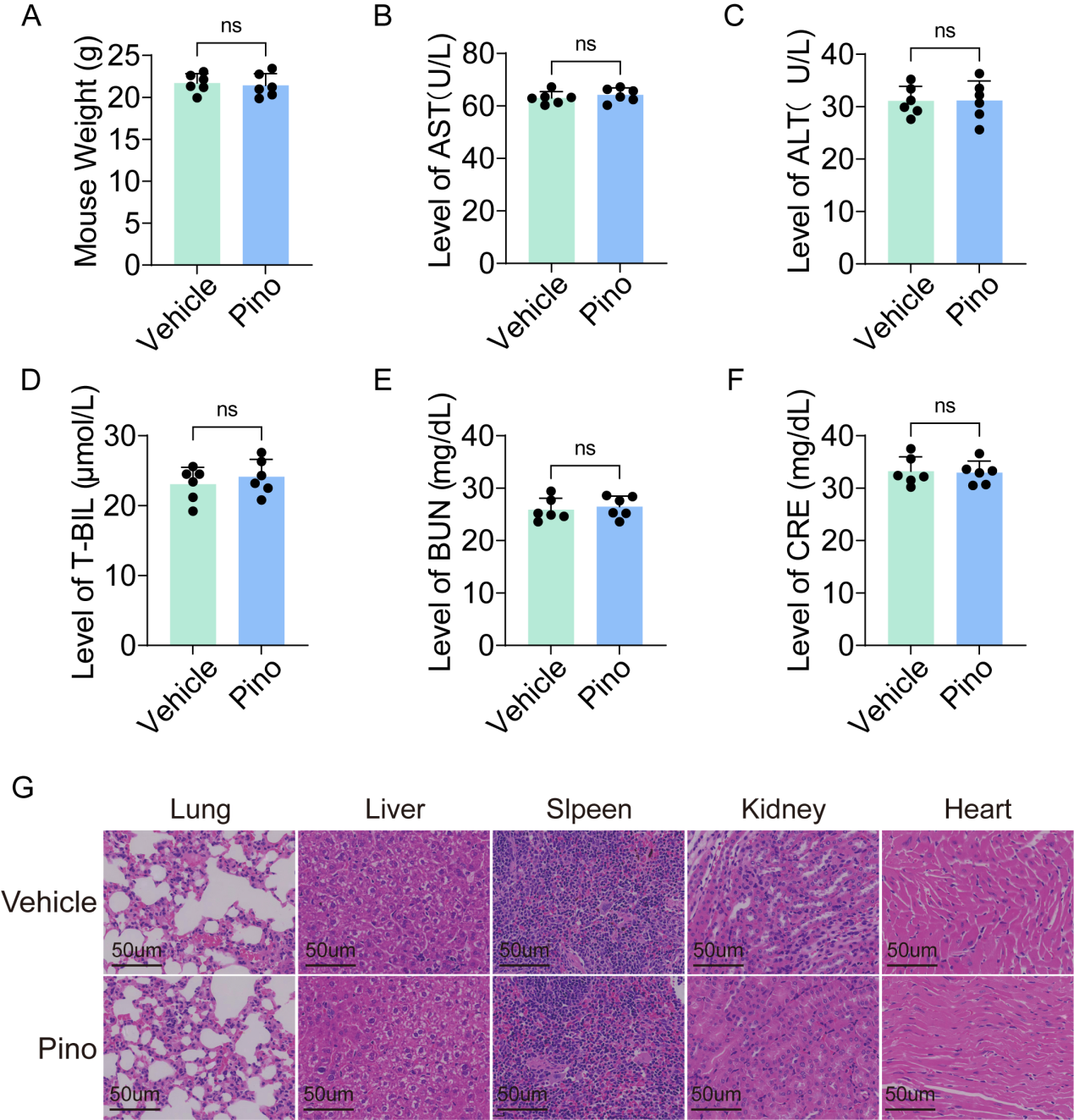


**Figure S6**

Intratumor injection administration of pinocembrin (pino) exhibited no obvious toxicity or adverse effects in mice. (A) Statistical comparison of body weights between vehicle and pinocembrin groups of mice. (B-D) Liver function in vehicle and pinocembrin groups was assessed by measuring serum levels of AST (B), ALT (C), and T-BIL(D). Kidney function was evaluated based on BUN (E) and CRE (F) levels. (G) Representative hematoxylin and eosin (H&E) staining images of the lung, liver, spleen, kidney, and heart from mice in the vehicle and pinocembrin groups. ns: not significant. AST: aspartate aminotransferase; ALT: alanine aminotransferase; T-BIL: total bilirubin; BUN: blood urea nitrogen; CRE: serum creatinine.


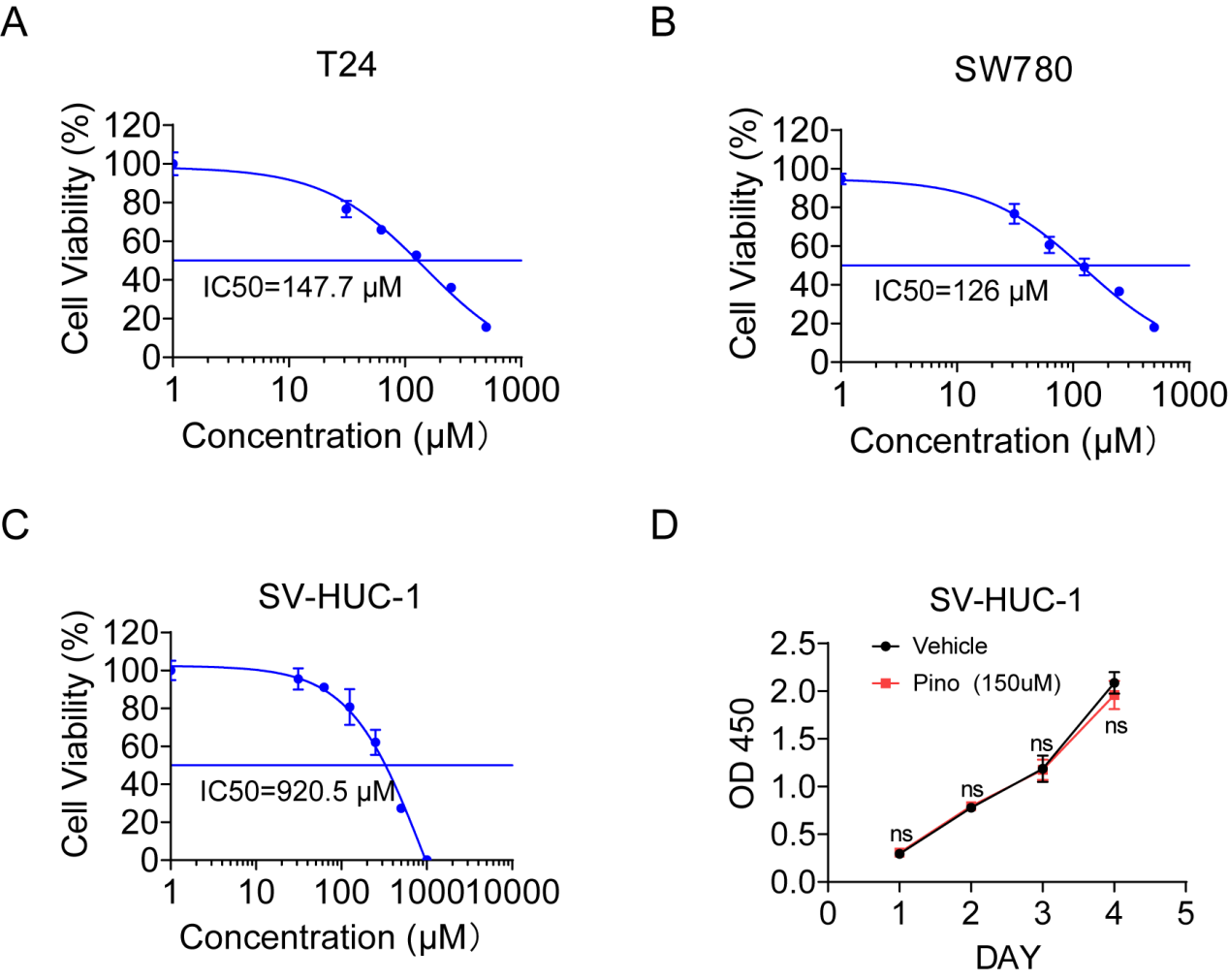


**Figure S7**

Normal epithelial cell of urothelium (SV-HUC-1) is more resistant to pinocembrin. (A-B) IC50 of pinocembrin in T24 and SW780 cells. (C) The IC50 of pinocembrin in SV-HUC-1. (D) The OD450 of SV-HUC-1.


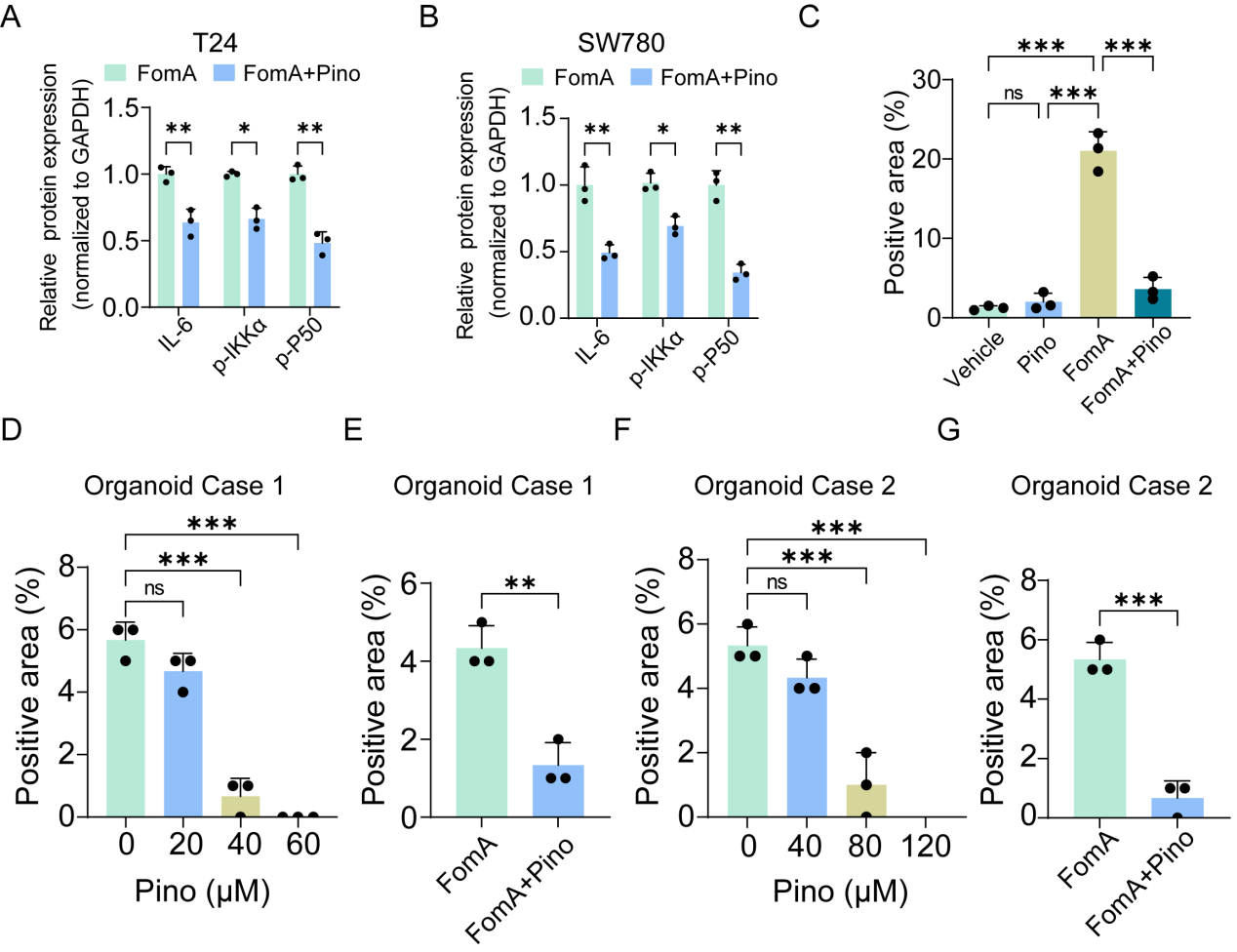


**Figure S8**

(A-B) Densitometric quantification of Western blot images (Fig 8F) showing the expression of TLR2, NF-κB1, and IL-6 in T24 and SW780 cells.

(C) Quantitative analysis of immunohistochemical (IHC) staining (Fig 8O) of lymph nodes from mice under the indicated treatment conditions.

(D-G) Quantitative analysis of proliferation (Fig 8P-S) in patient-derived bladder cancer organoids under the indicated treatment conditions.
